# Supplementary figures and images for: Unveiling the regulatory network controlling natural transformation in lactococci
Source: PLoS Genet. 2024 Jul 1;20(7):e1011340. doi: 10.1371/journal.pgen.1011340 (PMC11244767; doi:10.1371/journal.pgen.1011340)

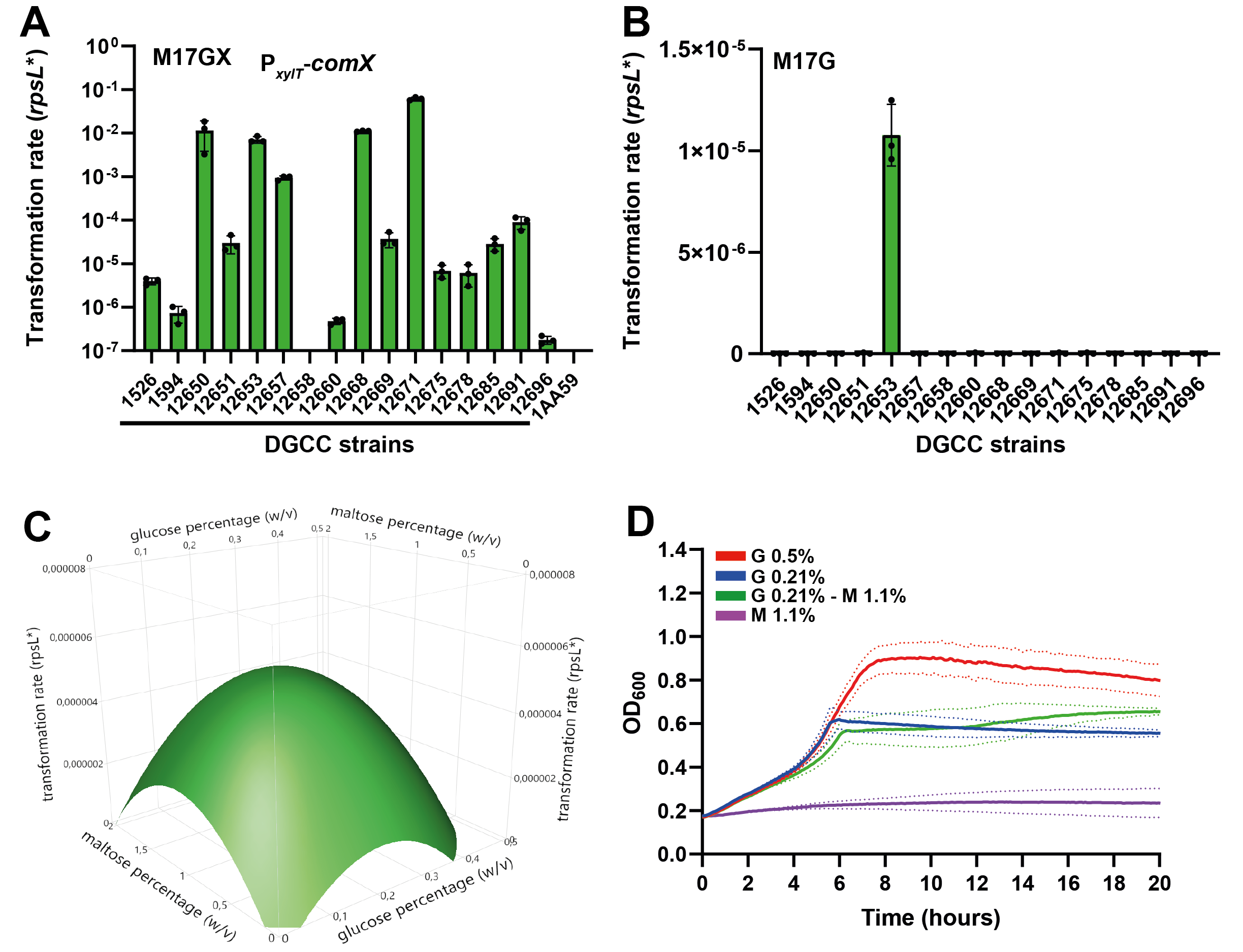

Supplement: S1 Fig — (A) Transformability upon ComX overexpression. Data show transformation rates of 16 DGCC strains and strain 1AA59 (negative control) harboring the pGhPxylT-comX plasmid in M17GX (0.1% glucose-1% xylose). All transformation assays were performed with rpsL* as donor DNA (20 μg ml-1), added at time zero. Cells were spread after ~24 hours of culture. Dots show the values for technical replicates (n = 3) ± standard deviations. (B) Transformability of 18 DGCC strains in M17G medium. Conditions for transformation assays as in panel A. Dots show the values for technical replicates (n = 3) ± standard deviations. (C) Effects of glucose-maltose combinations on spontaneous transformation of strain DGCC12653 WT in CDM. A design of experiment (composite central plan) was setup using the JMP Pro software. Sugar concentrations were varied from 0 to 2% (w/v) in 12 different conditions (glucose %/Maltose %; 0.01/0, 0.05/0.1, 0.3/0.1, 0/0.25, 0.2/0.25, 0.1/0.5, 0.5/0.5, 0.05/1.0, 0.3/1.0, 0/2.0, 0.2/2.0, and 0.4/2.0) containing donor DNA. Compilation of transformation rates was treated with JMP Pro and a response surface was generated. (D) Growth curves (OD600) of strain DGCC12653 WT in CDM-DEB supplemented with glucose 0.5% [G 0.5%], glucose 0.21% [G 0.21%], maltose 1.1% [M 1.1%], and a mixture of glucose 0.21% and maltose 1.1% [G0.21%-M 1.1%]. (TIF) [file pgen.1011340.s001.tif]

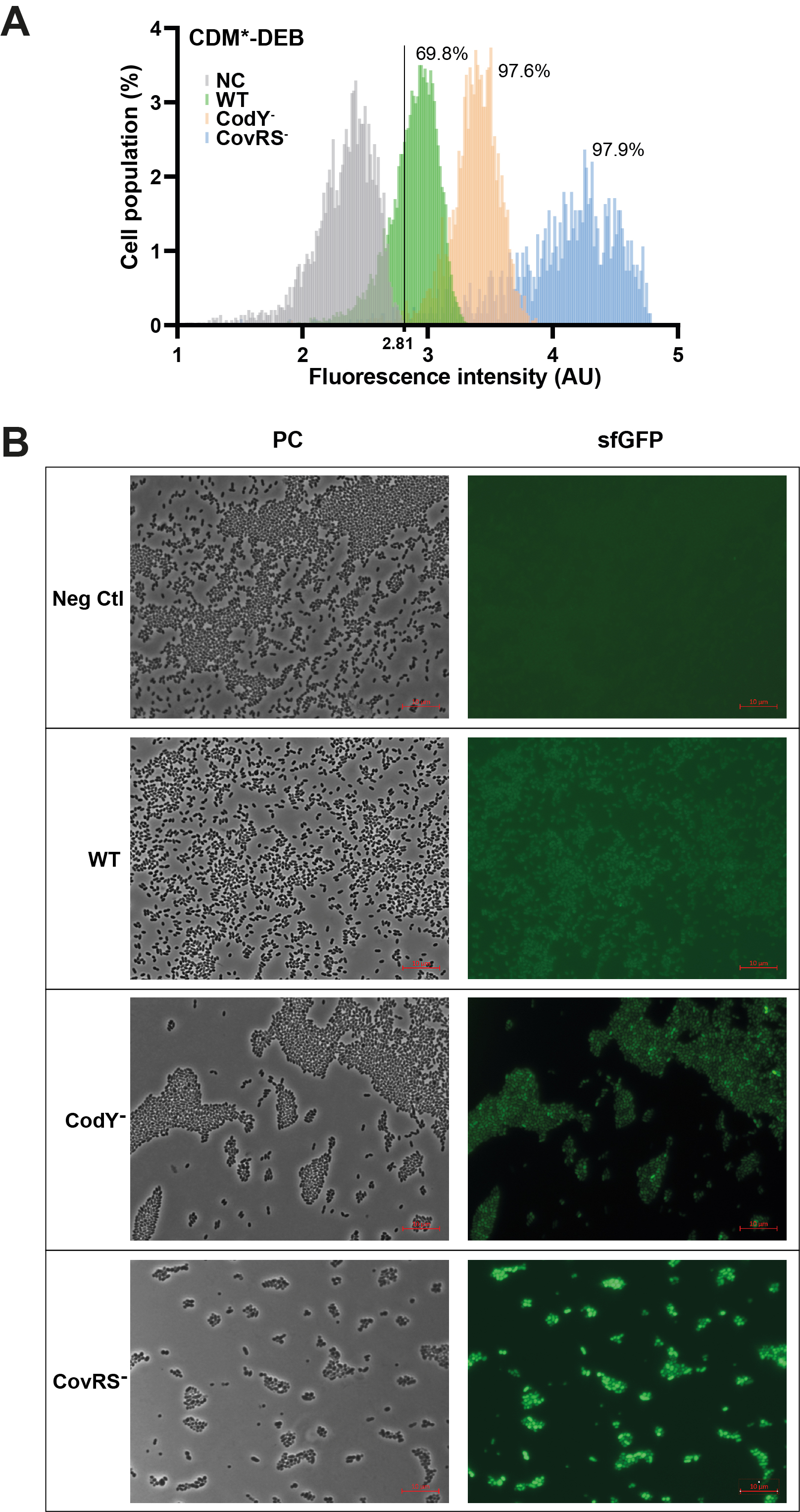

Supplement: S2 Fig — (A) Density plot of single-cell fluorescence intensity (arbitrary unit [AU]) for WT, CodY-, CovRS- strains harboring the reporter fusion PcomX-gfpsf (green, orange and blue, respectively) and the negative control (NC) harboring the empty plasmid (gray). Cells were cultured in CDM*-DEB, harvested at the diauxic shift, and analyzed by epifluorescence microscopy. The fluorescence of more than 1,200 individual cells was examined in each experiment. A minimum threshold of significant fluorescence (2.81 AU) was determined as the maximum signal reached by the negative control. Percentages in the top of the plot indicate the percentage of the cell population displaying a fluorescence signal higher than 2.81 for each strain. (B) Representative images of experiments depicted in panel A for two channels (left, phase contrast [PC] and right, sfGFP signal). (JPG) [file pgen.1011340.s002.jpg]

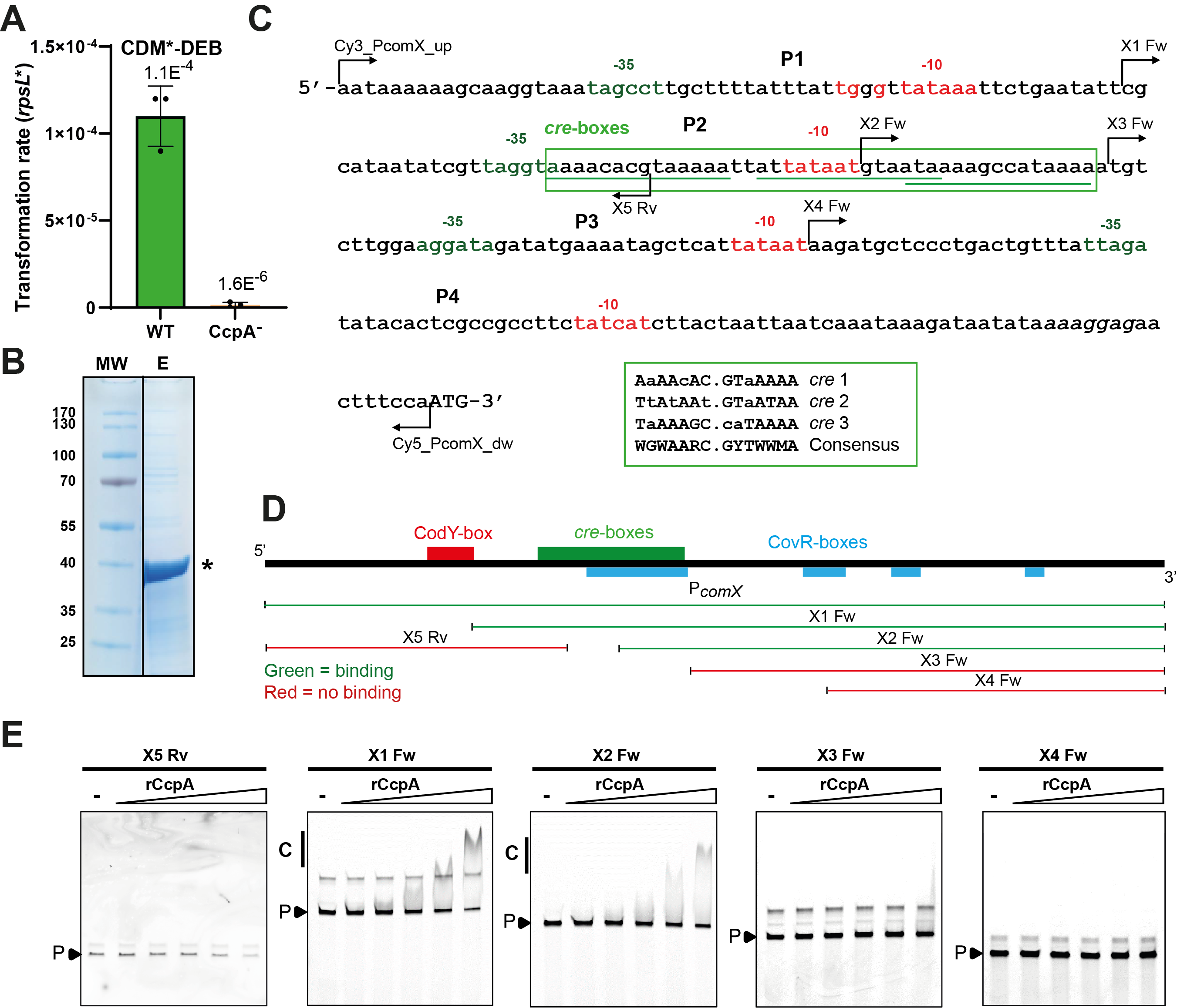

Supplement: S3 Fig — (A) Effect of ccpA deletion on transformability. Data show transformation rates observed for WT (green) and CcpA- (light brown) strains in CDM*-DEB after overnight culture with donor DNA. Transformation assays were performed with rpsL* as donor DNA (20 μg ml-1), added at time zero. Cells were spread after ~24 hours of culture. Dots show the values for biological triplicates (CcpA-) or technical triplicates (WT), mean values ± standard deviations. The mean value is indicated on the top each bar. (B) CcpA purification. SDS-PAGE of the elution step [E] of 6His-CcpA (rCcpA) purified from E. coli. MW, molecular weight (kDa). The star indicates the enriched rCcpA. (C) Mapping of fluorescent (Cy3_PcomX_up, Cy5_PcomX_dw) and non-fluorescent (X5 Rv, X1 Fw, X3 Fw, X2 Fw, X3 Fw, and X4 Fw) primers designed in PcomX (complete intergenic region). The region containing three cre-boxes (underlined) is surrounded in green. The alignment of the three cre-boxes with the consensus is shown with conserved positions in capitals. (D) Mapping of the different probes used for EMSAs. Green and red lines indicate the presence or absence of a band shift. (E) EMSAs performed with a gradient of purified CcpA (rCcpA) on the different probes shown in panel D. Lanes without rCcpA are indicated by a minus sign. C and P indicate the CcpA-DNA complex(es) and the unbound probe, respectively. (TIF) [file pgen.1011340.s003.tif]

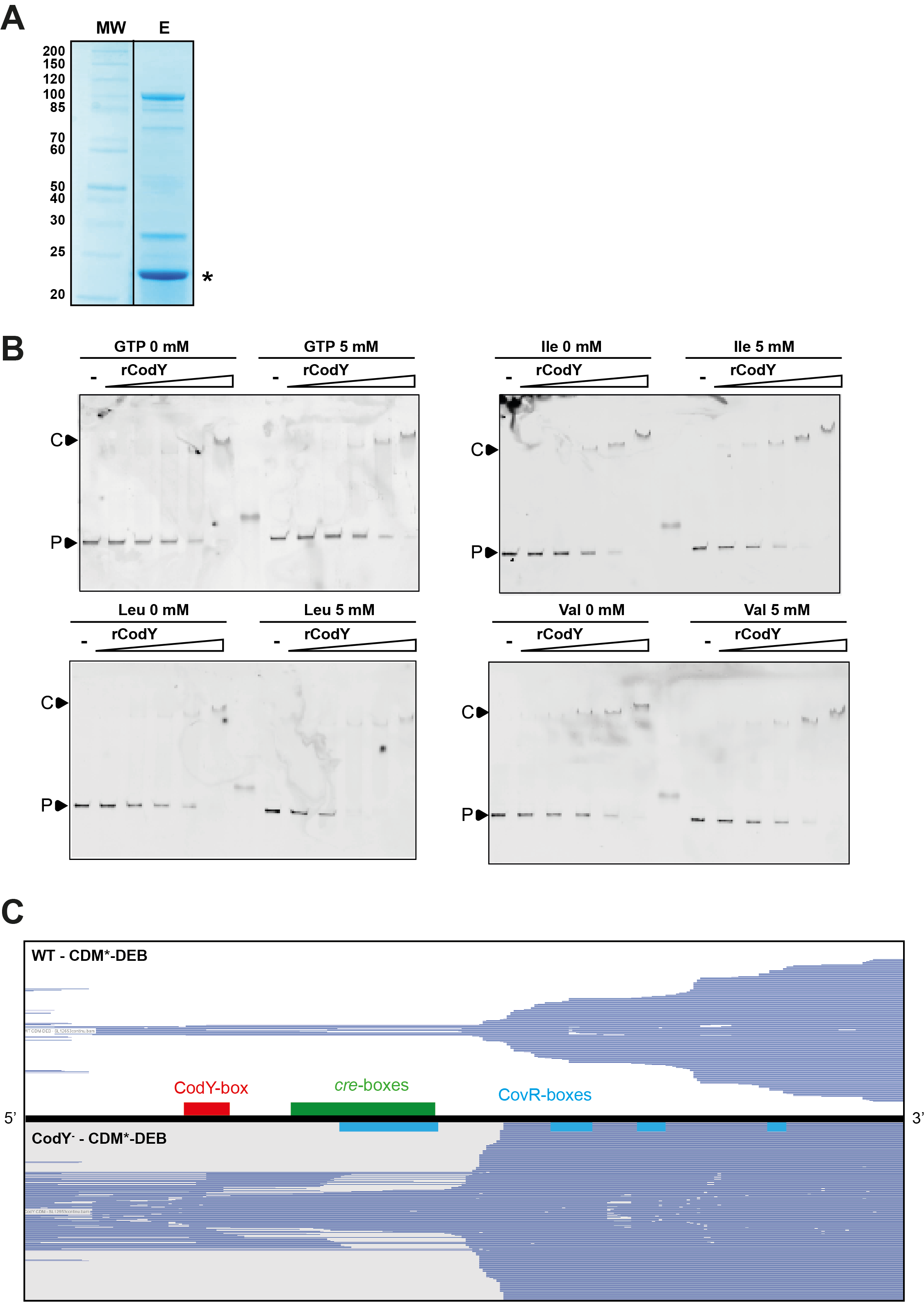

Supplement: S4 Fig — (A) CodY purification. SDS-PAGE of the elution step [E] of the 6His-CodY (rCodY) purified from L. lactis. MW, molecular weight (kDa). The star indicates the enriched rCodY. (B) EMSAs performed with a gradient of purified CodY (rCodY) on PcomX in absence (left side of each panel) or presence (right side of each panel) of 5 mM of GTP (top left panel), Ile (top right panel), Leu (lower left panel) or Val (lower right panel). Lanes without rCodY are indicated by a minus sign. C and P indicate rCodY-PcomX complex and unbound probe, respectively. (C) Mapping of RNAseq reads on PcomX, from WT (top panel) and CodY- (lower panel) strains. Cells were harvested at the diauxic shift in CDM*-DEB. The PcomX (complete intergenic region) is illustrated as a black line, while CodY-, cre- and CovR-boxes are localized and displayed in red, green and blue, respectively. (TIF) [file pgen.1011340.s004.tif]

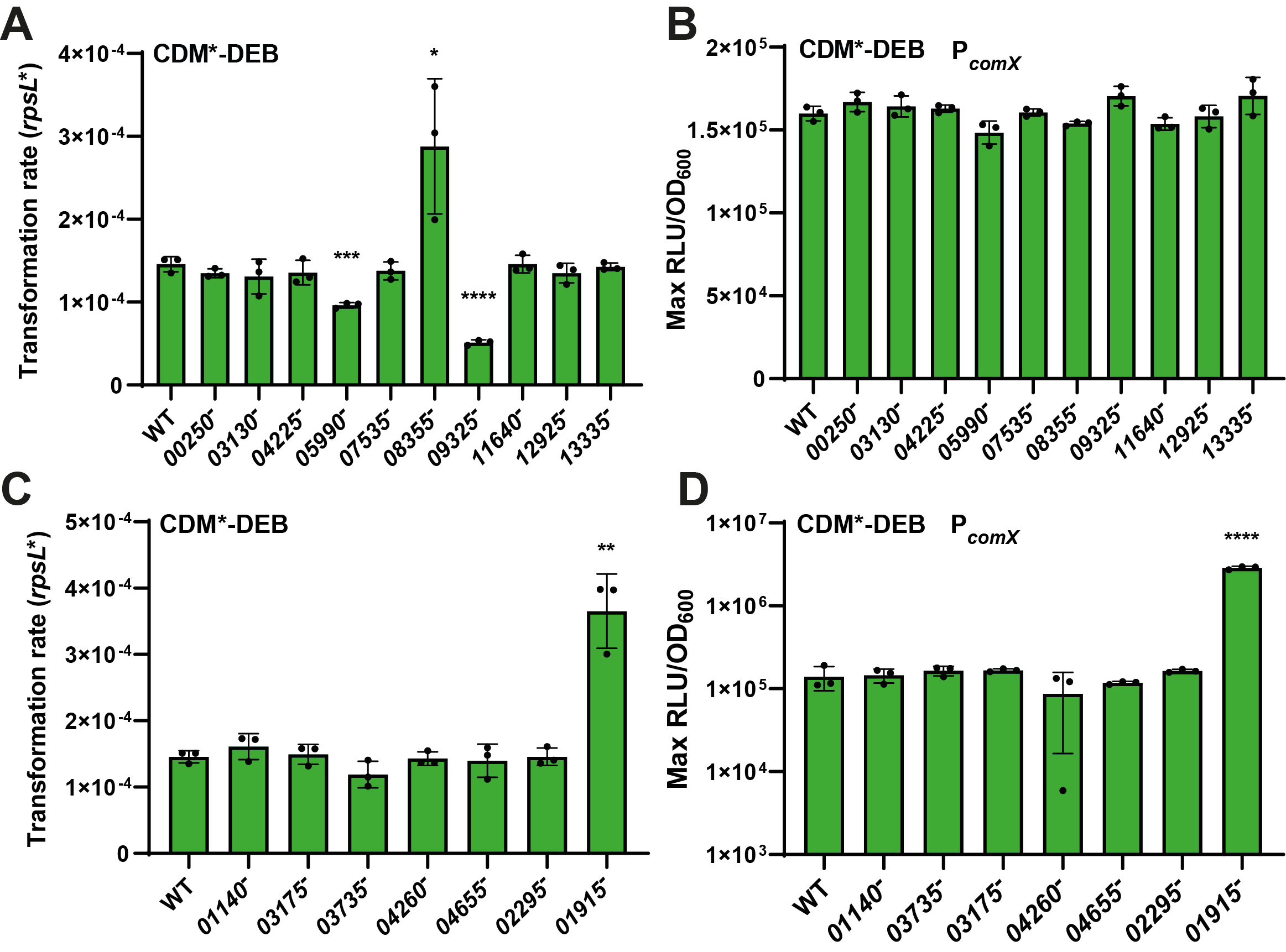

Supplement: S5 Fig — (A and B) Effects on transformability (A) and PcomX activity (B) of the independent inactivation of 10 Rgg from DGCC12653. (C and D) Effects on transformability (C) and PcomX activity (D) of the independent inactivation of 7 TCS from DGCC12653 (labels indicate the first gene of the TCS). Data on transformability (panels A and C) show transformation rates observed for WT and mutant strains in CDM*-DEB after overnight culture with donor DNA. Transformation assays were performed with rpsL* as donor DNA (20 μg ml-1), added at time zero. Cells were spread after ~24 hours of culture. Data on PcomX activity (panels B and D) show maximum specific luciferase activity (Max RLU/OD600) observed at the diauxic shift in CDM*-DEB. Dots (panels A to D) show the values for biological triplicates (Rgg- and TCS- strains) or technical triplicates (WT), mean values ± standard deviations. Statistical t test was performed for each mutant strain in comparison to the WT (n = 3; *, P < 0.05; **, P < 0.01; ***, P < 0.001; ****, P < 0.0001). (TIF) [file pgen.1011340.s005.tif]

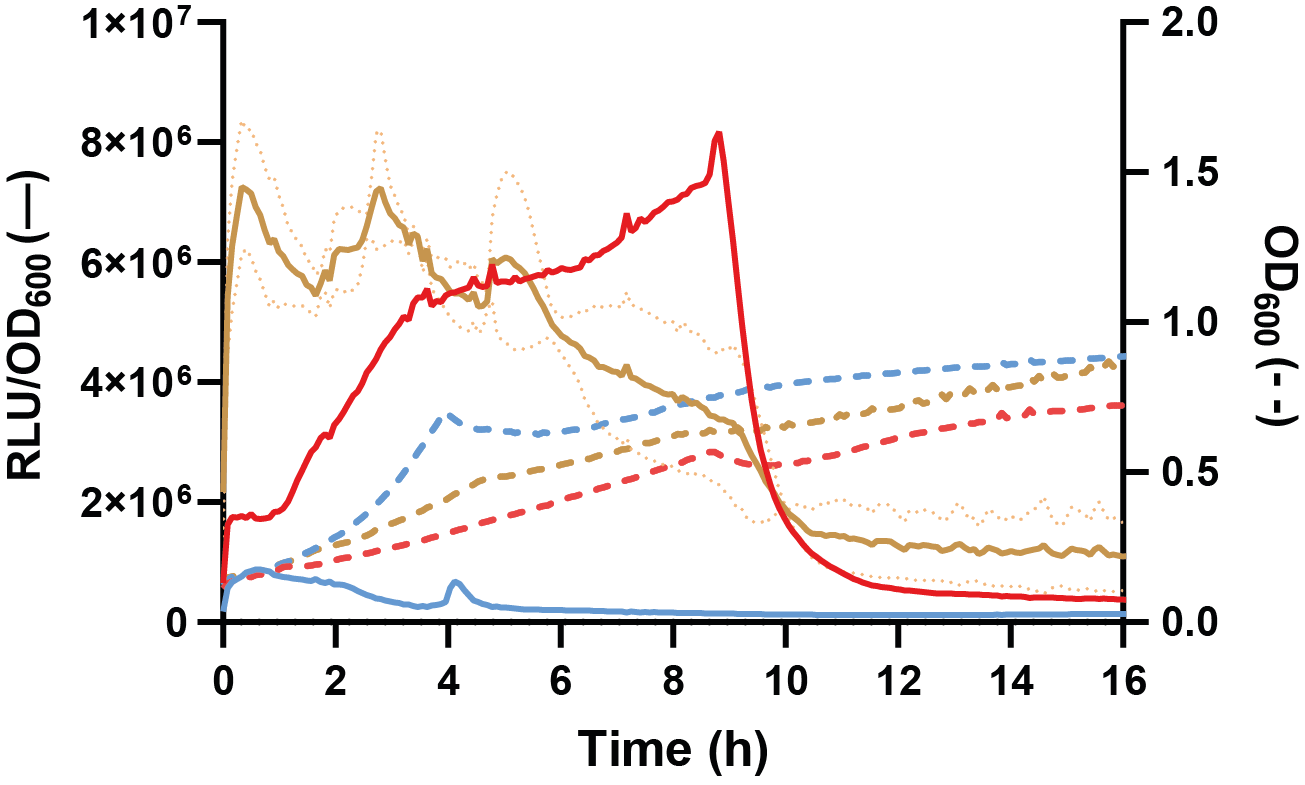

Supplement: S6 Fig — Growth (dotted lines–OD600) and kinetics of PcomX specific luciferase activity (continuous lines–RLU/OD600) monitored over time for CodY- (blue), CovRS- (red) and CodY- CovRS- (brown) mutant strains. Continuous and dotted dark lines are the mean values of biological triplicates, and light lines are standard deviations. (TIF) [file pgen.1011340.s006.tif]

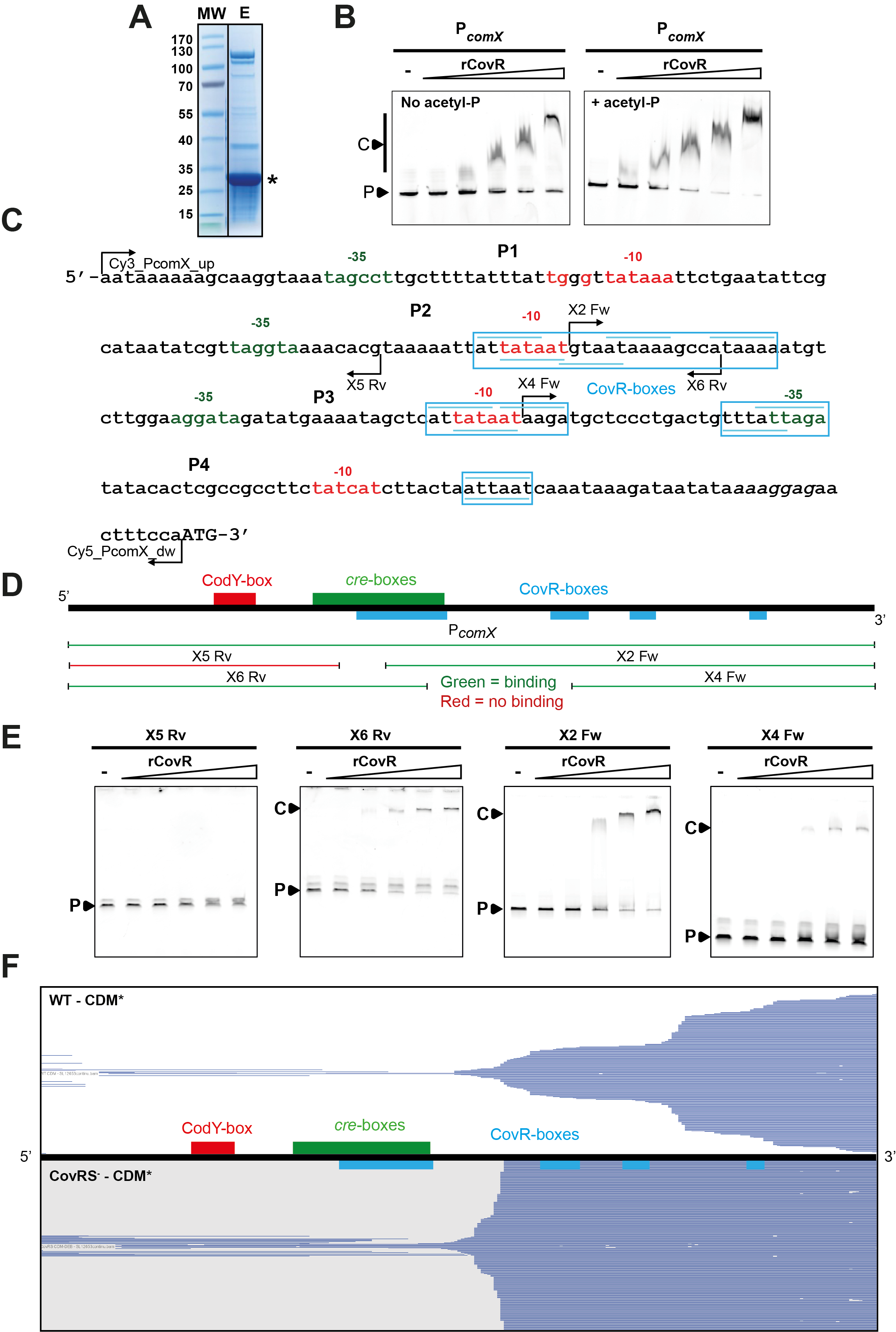

Supplement: S7 Fig — (A) CovR purification. SDS-PAGE of the elution step [E] of the 6His-CovR (rCovR) purified from L. lactis. MW, molecular weight (kDa). The star indicates the enriched rCovR. (B) EMSAs performed with a gradient of purified CovR (rCovR) without (left) or with (right) in vitro phosphorylation with acetyl-P on PcomX. Lanes without rCovR are indicated by a minus sign. C and P indicate the rCovR-DNA complex(es) and the unbound probe, respectively. (C) Mapping of fluorescent (Cy3_PcomX_up, Cy5_PcomX_dw) and non-fluorescent (X5 Rv, X6 Rv, X2 Fw, X3 Fw, and X4 Fw) primers designed in PcomX (complete intergenic region). The regions containing CovR-boxes (underlined) are surrounded in blue. (D) Mapping of the different probes used for EMSAs. Green and red lines indicate the presence or absence of a band shift. (E) EMSAs performed with a gradient of purified CovR (rCovR) on the different probes shown in panel C. Lanes without rCovR are indicated by a minus sign. C and P indicate the rCovR-DNA complex(es) and the unbound probe, respectively. (F) Mapping of RNAseq reads on PcomX, from WT (top panel) and CovRS- (lower panel) strains. Cells were harvested at the diauxic shift in CDM*. The PcomX (complete intergenic region) is illustrated as a black line, while CodY-, cre- and CovR-boxes are localized and displayed in red, green, and blue, respectively. (TIF) [file pgen.1011340.s007.tif]

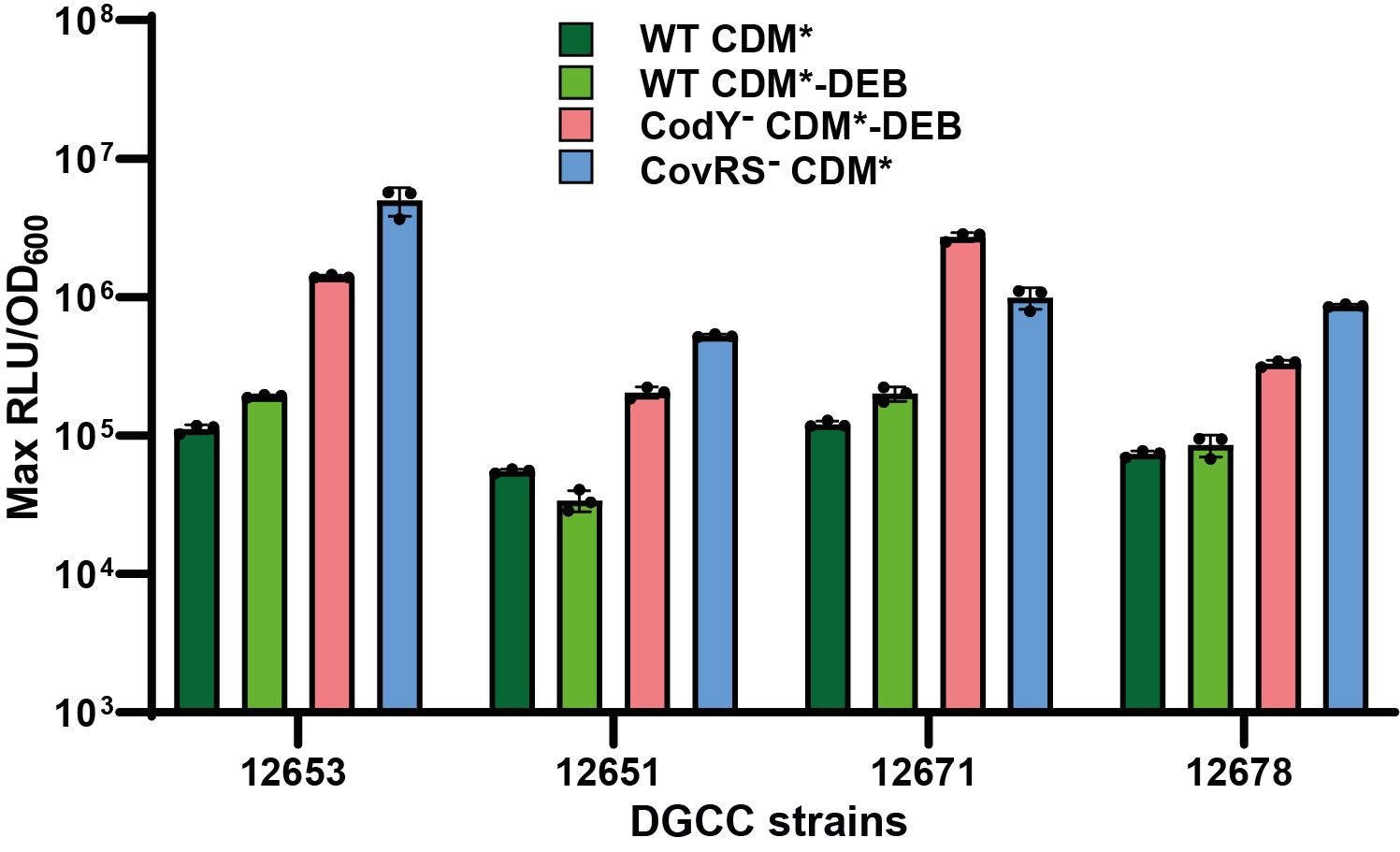

Supplement: S8 Fig — The effects of these deletions (CodY- in CDM*-DEB, light red bars and CovRS- in CDM*, blue bars) were compared to their respective WT in the same culture conditions (WT in CDM*, dark green bars and CDM*-DEB, light green bars). Data show maximum specific luciferase activity (Max RLU/OD600) observed at the diauxic shift either in CDM*-DEB or CDM*. Dots show the values for biological triplicates (CodY- and CovR- strains) or technical triplicates (WT), mean values ± standard deviations. (TIF) [file pgen.1011340.s008.tif]

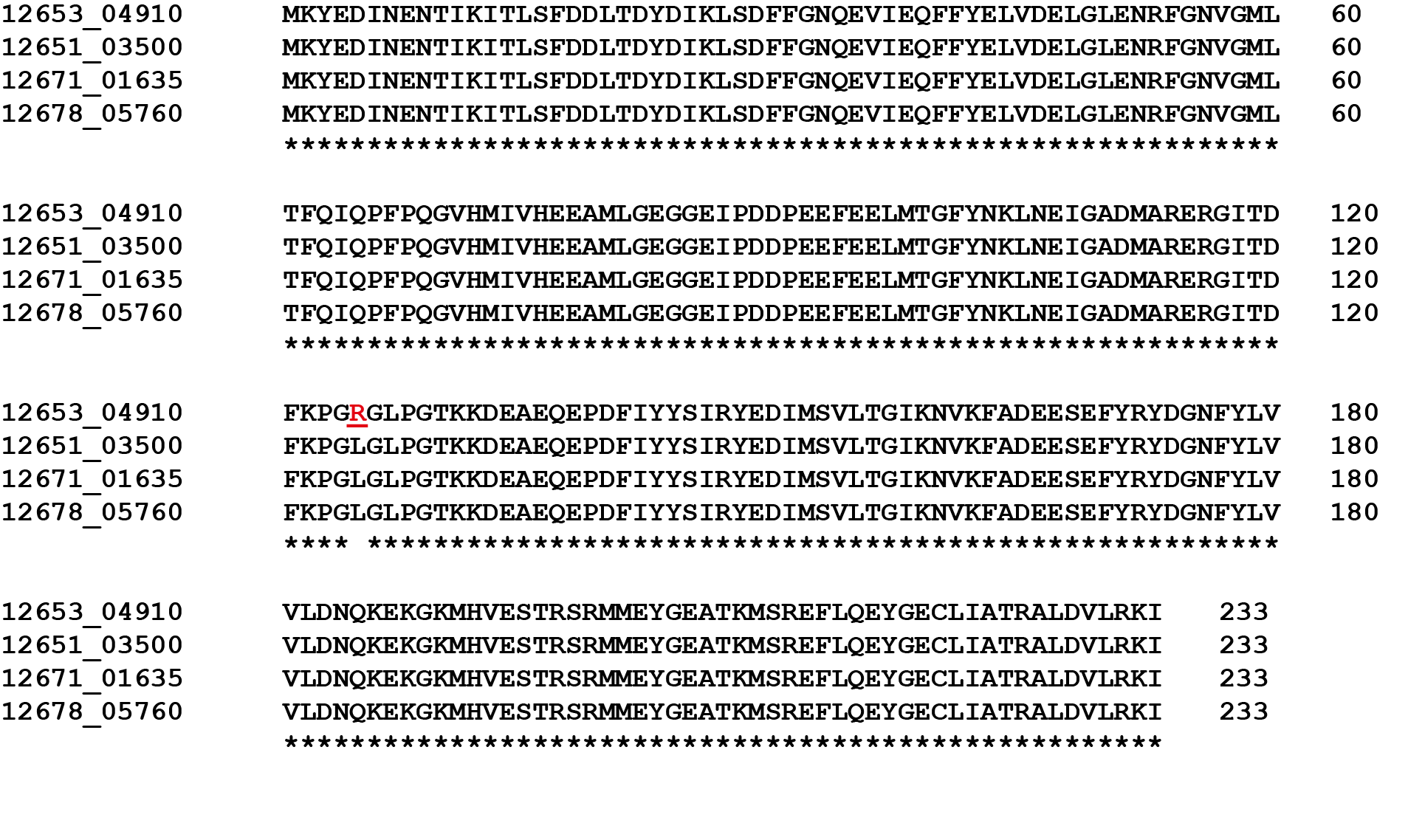

Supplement: S10 Fig — The L125R substitution is in red and underlined in MecA from strain DGCC12653. Strains DGCC12651, DGCC12671 and DGCC12678 contain the common MecA-L125 residue. The alignment was performed using Clustal Omega. (TIF) [file pgen.1011340.s010.tif]
