# Supplementary material for: Unveiling the regulatory network controlling natural transformation in lactococci
Source: PLoS Genet. 2024 Jul 1;20(7):e1011340. doi: 10.1371/journal.pgen.1011340 (PMC11244767; doi:10.1371/journal.pgen.1011340)
Supplement: S1 Table — (PDF) [file pgen.1011340.s011.pdf]

S1 Table. List of key genes for DNA transformation from the ComX regulon

Locus tag DGCC12653	Locus tag KF147	Gene[a]	Description
**Regulation**			
DGCC12653_13390	LLKF_2393	*comX*	Competence-specific transcriptional regulator
**Pilus biogenesis**			
DGCC12653_09995	LLKF_2372	*comGA*	Competence type IV pilus, ATPase
DGCC12653_10000	LLKF_2371	*comGB*	Competence type IV pilus, assembly protein
DGCC12653_10005	LLKF_2370	*comGC*	Competence type IV pilus, major pilin
DGCC12653_10010	LLKF_2369	*comGD*	Competence type IV pilus, minor pilin
DGCC12653_10015	LLKF_2368	*comGE*	Competence type IV pilus, minor pilin
DGCC12653_10020	LLKF_2367	*comGF*	Competence type IV pilus, minor pilin
DGCC12653_10025	LLKF_2366	*comGG*	Competence type IV pilus, minor pilin
DGCC12653_09640	LLKF_2214	*comC*	Type IV prepilin peptidase
**DNA uptake**			
DGCC12653_04795	LLKF_1945	*comEA*	DNA uptake machinery, DNA receptor
DGCC12653_04790	LLKF_1944	*comEC*	DNA uptake machinery, DNA uptake channel
DGCC12653_02995	LLKF_1117	*comFA*	DNA uptake machinery, DNA transporter ATPase
DGCC12653_03000	LLKF_1116	*comFC*	DNA uptake machinery, phosphorybosyltransferase domain
**DNA protection and recombination**			
DGCC12653_06395	LLKF_0444	*ssbB*	ssDNA binding protein
DGCC12653_02130	LLKF_1273	*dprA*	DNA recombination-mediator protein A, RecA loader
DGCC12653_04550	LLKF_1899	*coiA*	Competence protein, nuclease domain
DGCC12653_06225	LLKF_0409	*recA*	Recombinase A protein
**Transformasome**			

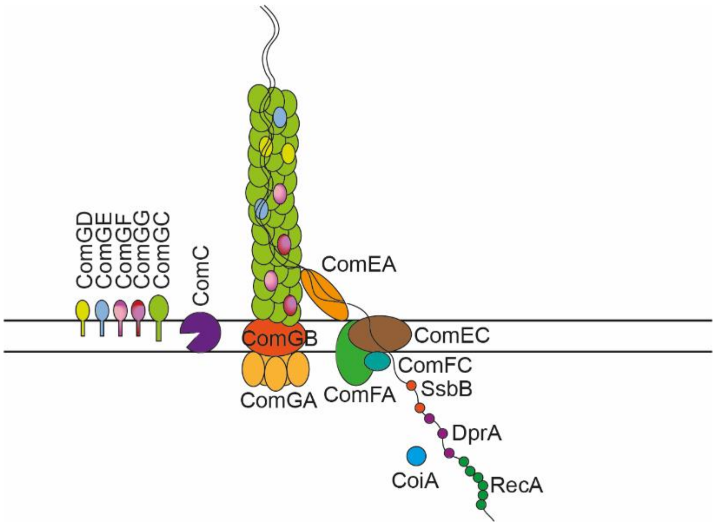

[a] The set of key *com* genes from the ComX regulon was selected from *Streptococcus pneumoniae* [1] and latter shown to be under the control of ComX in *Lactococcus cremoris* [2]. A scheme at the bottom display their products for pilus biogenesis, DNA uptake, DNA protection, and DNA recombination [3].

References

1. Peterson S, Cline RT, Tettelin H, Sharov V, Morrison DA. Gene expression analysis of the *Streptococcus pneumoniae* competence regulons by use of DNA microarrays. J Bacteriol. 2000 Nov; 182(21):6192-202. 0552 [pii];10.1128/JB.182.21.6192-6202.2000 [doi].

2. David B, Radziejwoski A, Toussaint F, Fontaine L, de Frahan MH, Patout C et al. Natural DNA Transformation Is Functional in *Lactococcus lactis* subsp. *cremoris* KW2. Appl Environ Microbiol. 2017 Aug 15; 83(16). AEM.01074-17 [pii];01074-17 [pii];10.1128/AEM.01074-17 [doi].

3. Di Giacomo S., Toussaint F, Ledesma-Garcia L, Knoops A, Vande CF, Fremaux C et al. Expanding natural transformation to improve beneficial lactic acid bacteria. FEMS Microbiol Rev. 2022 Jul 20; 46(4). 6543703 [pii];fuac014 [pii];10.1093/femsre/fuac014 [doi].
