# Supplementary material for: Unveiling the regulatory network controlling natural transformation in lactococci
Source: PLoS Genet. 2024 Jul 1;20(7):e1011340. doi: 10.1371/journal.pgen.1011340 (PMC11244767; doi:10.1371/journal.pgen.1011340)
Supplement: S2 Table — (PDF) [file pgen.1011340.s012.pdf]

S2 Table. Up-regulation of the ComX regulon analyzed by RNA sequencing [a]

Locus_tag SL12653_#	Locus_tag KF147	Gene symbol	Strand	Function	$\frac{\Delta covRS}{WT}$	$\frac{\Delta codY}{WT}$	$\frac{PxylTcomX}{WT}$	ComX-box (5'-3') [b]
**Competence regulation**								
13390	LLKF_2393	*comX*	+	Competence-specific transcriptional regulator	5.97	8.19	194.97	
**Pseudopilus biogenesis**								
9995	LLKF_2372	*comGA*	+	Competence type IV pilus, ATPase	3305.56	23.61	21999.94	gttacattaactgattatttgcgtata
10000	LLKF_2371	*comGB*	+	Competence type IV pilus, assembly protein	1327.45	23.27	50327.56	
10005	LLKF_2370	*comGC*	+	Competence type IV pilus, major pilin	622.61	33.48	5703.10	
10010	LLKF_2369	*comGD*	+	Competence type IV pilus, minor pilin	146.84	23.70	3722.97	
10015	LLKF_2368	*comGE*	+	Competence type IV pilus, minor pilin	251.91	16.25	8049.18	
10020	LLKF_2367	*comGF*	+	Competence type IV pilus, minor pilin	542.33	29.15	12178.76	
10025	LLKF_2366	*comGG*	+	Competence type IV pilus, minor pilin	18.93	39.06	580.95	
9640	LLKF_2214	*comC*	+	Type IV prepilin peptidase	209.62	19.48	538.53	gttacaattcttcatttatttcgtata
**DNA uptake machinery**								
4790	LLKF_1944	*comEC*	-	DNA uptake machinery, DNA uptake channel	183.82	19.18	1711.63	
4795	LLKF_1945	*comEA*	-	DNA uptake machinery, DNA receptor	336.76	16.95	1202.00	gaaactttccaagagtttttttcgtata
2995	LLKF_1117	*comFA*	+	DNA uptake machinery, DNA transporter ATPase	354.24	28.93	1814.76	gttacattttaacagcattttcgtata
3000	LLKF_1116	*comFC*	+	DNA uptake machinery, phosphorybosyltransferase domain	2362.52	29.16	2872.44	
**DNA protection and recombination**								
06395	LLKF_0444	*ssbB*	+	ssDNA binding protein	178.41	17.40	1092.40	gtgacaaatgtcctaactttacgtata
06400	LLKF_0445	*groES*	+	Heat shock protein 60 family co-chaperone GroES	5.13	7.69	39.77	
06405	LLKF_0446	*groEL*	+	Heat shock protein 60 family chaperone GroEL	3.17	6.06	38.22	
02125	LLKF_1274	*topA*	-	DNA topoisomerase I	15.47	3.99	55.36	
02130	LLKF_1273	*dprA*	-	DNA recombination-mediator protein A, RecA loader	683.19	26.98	19633.07	gttacaagtcagtctttttttacgtata
04545	LLKF_1898	*pepF*	-	Oligoendopeptidase F	3.69	1.78	16.97	

04550	LLKF_1899	*coiA*	-	Competence protein, nuclease domain	21.59	16.35	38.87	gttacaaataaatactttttttcgtata
06225	LLKF_0409	*recA*	+	Recombinase A protein	3.16	2.60	12.58	gtgacaaagttaataaaaaaaacgtata
03150	LLKF_1029	*radC*	-	JAB domain-containing protein	220.72	18.07	1478.37	gtgacaaaggccagattttttccgtata
**Other functions**								
11355	LLKF_1769	*yqfG*	+	Hypothetical extracellular protein, C-terminal membrane anchor	560.61	29.84	1801.77	gttacaaaacctccatttttttcgtata
11360	LLKF_1768	*ffh*	+	Signal recognition particle, subunit Ffh SRP54	8.21	3.81	32.68	
05985	LLKF_0364	*ydbC*	+	Hypothetical protein, DNA-binding domain	3.82	3.27	13.67	gtgacaaaagtgatttattttcgtata
05995	LLKF_0366		+	Hypothetical protein	17.26	2.47	3.16	
06000	LLKF_0367		+	ABC transporter, ATP binding and permease	9.30	27.70	1.80	
**ComX box**								

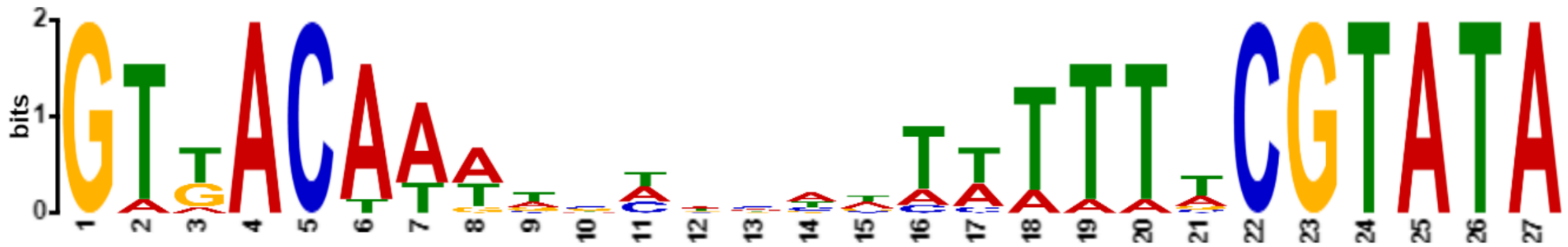

[a] Up-regulated genes of the ComX regulon correspond to genes with a 3-fold increase in the CovRS-deficient strain compared to the wild type and preceded by a ComX-box. Transcriptional units are separated by a dark line.

[b] The ComX box (5'-GTKACAAWWNHWNWWTWTTTWCGTATA-3'), previously identified in *L. lactis* and *L. cremoris* [1–3], was used. The identification of ComX boxes was performed with the MEME suite (https://meme-suite.org/meme/). The logo of the ComX box generated from boxes reported in this table is displayed at the bottom.

References

1. David B, Radziejwoski A, Toussaint F, Fontaine L, de Frahan MH, Patout C et al. Natural DNA Transformation Is Functional in *Lactococcus lactis* subsp. *cremoris* KW2. Appl Environ Microbiol. 2017 Aug 15; 83(16). AEM.01074-17 [pii];01074-17 [pii];10.1128/AEM.01074-17 [doi].

2. Mulder J, Wels M, Kuipers OP, Kleerebezem M, Bron PA. Unleashing Natural Competence in *Lactococcus lactis* by Induction of the Competence Regulator ComX. Appl Environ Microbiol. 2017 Oct 15; 83(20). AEM.01320-17 [pii];01320-17 [pii];10.1128/AEM.01320-17 [doi].

3. Wydau S, Dervyn R, Anba J, Dusko ES, Maguin E. Conservation of key elements of natural competence in *Lactococcus lactis* ssp. FEMS Microbiol Lett. 2006 Apr; 257(1):32-42. FML141 [pii];10.1111/j.1574-6968.2006.00141.x [doi].
