# Supplementary material for: Unveiling the regulatory network controlling natural transformation in lactococci
Source: PLoS Genet. 2024 Jul 1;20(7):e1011340. doi: 10.1371/journal.pgen.1011340 (PMC11244767; doi:10.1371/journal.pgen.1011340)
Supplement: S3 Table — (PDF) [file pgen.1011340.s013.pdf]

**S3 Table. Annotation of TCS and Rgg sensors from *L. lactis* DGCC12653**

| | Locus tag DGCC12653_# | Locus tag KF147 | Locus tag IL1403 | Occurrence in 18 DGCC strains[a] | Initial gene name[b] | Revised gene name | Known functions in closely related Gram-positive bacteria[c] |
|---|---|---|---|---|---|---|---|
| Two-Component Systems | 01915 | LLKF_1744 | L0131 | 18 / 0 / 0 | *llrA* | *covR* | Acid resistance and arginine metabolism in *L. cremoris* [1]. Control of virulence in pathogenic streptococci [2]. Control of competence/predation in salivarius streptococci [3] |
| | 01910 | LLKF_1743 | L0130 | 18 / 0 / 0 | | *covS* | |
| | 01140 | LLKF_1540 | L0123 | 18 / 0 / 0 | *llrB* | *phoP* | Control of phosphate homeostasis in bacilli [4] |
| | 01145 | LLKF_1541 | L0122 | 17 / 1 / 0 | | *phoR* | |
| | 06420 | LLKF_0449 | L0125 | 18 / 0 / 0 | *llrC* | *walR/vicR* | Control of cell division and cell wall homeostasis [5–7] |
| | 06415 | LLKF_1024 | L0124 | 18 / 0 / 0 | | *walK/vicK* | |
| | 03735 | LLKF_0918 | L0127 | 18 / 0 / 0 | *llrD* | *cseR* | Response to salt/osmotic and envelope stresses in *L. cremoris* [1,8] |
| | 03740 | LLKF_0917 | L0126 | 18 / 0 / 0 | | *cseS* | |
| | 03175 | LLKF_1024 | L0129 | 18 / 0 / 0 | *llrE* | *llrE* | Control of phosphatase activity in *L. cremoris* [1] |
| | 03165 | LLKF_1026 | L0128 | 18 / 0 / 0 | | *kinE* | |
| | 04260 | LLKF_1834 | L0135 | 18 / 0 / 0 | *llrF* | *ciaR* | Response to oxidative stress in *L. cremoris* [1]. Repression of competence in *S. pneumoniae* [9] |
| | 04255 | LLKF_1833 | L0134 | 18 / 0 / 0 | | *ciaH* | |
| | 04655 | LLKF_1918 | L0133 | 18 / 0 / 0 | *llrG* | *braR/bceR* | Resistance to bacteriocins and antibiotics in *L. cremoris* [10,11] |
| | 04650 | LLKF_1917 | L0132 | 18 / 0 / 0 | | *braS/bceS* | |
| | 02295 | LLKF_1239 | NH[d] | 18 / 0 / 0 | *kdpED* | *kdpE* | Control of potassium transport and regulation of turgor pressure [12] |
| | 02300 | LLKF_1238 | NH | 18 / 0 / 0 | | *kdpD* | |
| Rgg sensors | 00250 | LLKF_1358 | L19959 | 18 / 0 / 0 | | *gadR* | Rgg regulator controlling glutamate-γ-aminobutyrate production and acid resistance in *L. cremoris* [13,14] |
| | 03130 | LLKF_1092 | NH | 11 / 1 / 6 | | *ykhI* | Unknown |
| | 04225[f] | LLKF_1827 | L162840 | 17 / 1 / 0 | | *yrbI* | Unknown |
| | 05990 | LLKF_0365 | L116756 | 13 / 5 / 0 | | *ywdE_bis* | Unknown |
| | 07535 | LLKF_0776 | L126656 | 18 / 0 / 0 | | *yhgC* | Unknown |
| | 08355 | LLKF_0051 | L77381 | 18 / 0 / 0 | | *yrbI_bis* | Unknown |
| | 09325 | NH | L70850 | 12 / 2 / 4 | | *yueB* | Unknown |

| | | | | | |
|---|---|---|---|---|---|
| 11640 | LLKF_2477 | L27433 | 15 / 3 / 0 | *ywiI* | Unknown |
| 12925 | LLKF_0034[d] | NH | 11 / 7 / 0 | *(kw2_0044)* | Unknown |
| 13335 | LLKF_2403 | NH | 12 / 0 / 6 | *ywdE* | Unknown |

[a] Numbers refer to the occurrence of intact/truncated/absent genes among the 18 selected DGCC strains of this work.
[b] Initial gene names of TCSs according to *Lactococcus cremoris* MG1363 [1].
[c] List of known functions in other Gram-positive bacteria is not exhaustive.
[d] NH, No Homolog.
[f] Inactive, misplaced start codon.
[d] Truncated, only N-terminal part.

## References


1. O'Connell-Motherway M, van SD, Morel-Deville F, Fitzgerald GF, Ehrlich SD, Morel P. Six putative two-component regulatory systems isolated from *Lactococcus lactis* subsp. *cremoris* MG1363. Microbiology (Reading ). 2000 Apr; 146 ( Pt 4)935-47. 10.1099/00221287-146-4-935 [doi].

2. Churchward G. The two faces of Janus: virulence gene regulation by CovR/S in group A streptococci. Mol Microbiol. 2007 Apr; 64(1):34-41. MMI5649 [pii];10.1111/j.1365-2958.2007.05649.x [doi].

3. Knoops A, Vande CF, Fontaine L, Verhaegen M, Mignolet J, Goffin P et al. The CovRS Environmental Sensor Directly Controls the ComRS Signaling System To Orchestrate Competence Bimodality in Salivarius Streptococci. mBio. 2022 Jan 4; 13e0312521. 10.1128/mbio.03125-21 [doi].

4. Salzberg LI, Botella E, Hokamp K, Antelmann H, Maass S, Becher D et al. Genome-wide analysis of phosphorylated PhoP binding to chromosomal DNA reveals several novel features of the PhoPR-mediated phosphate limitation response in *Bacillus subtilis*. J Bacteriol. 2015 Apr; 197(8):1492-506. JB.02570-14 [pii];02570-14 [pii];10.1128/JB.02570-14 [doi].

5. Dobihal GS, Brunet YR, Flores-Kim J, Rudner DZ. Homeostatic control of cell wall hydrolysis by the WalRK two-component signaling pathway in *Bacillus subtilis*. Elife. 2019 Dec 6; 8. 52088 [pii];10.7554/eLife.52088 [doi].

6. Wang S, Long L, Yang X, Qiu Y, Tao T, Peng X et al. Dissecting the Role of VicK Phosphatase in Aggregation and Biofilm Formation of *Streptococcus mutans*. J Dent Res. 2021 Jun; 100(6):631-8. 10.1177_0022034520979798 [pii];10.1177/0022034520979798 [doi].

7. Wayne KJ, Li S, Kazmierczak KM, Tsui HC, Winkler ME. Involvement of WalK (VicK) phosphatase activity in setting WalR (VicR) response regulator phosphorylation level and limiting cross-talk in *Streptococcus pneumoniae* D39 cells. Mol Microbiol. 2012 Nov; 86(3):645-60. 10.1111/mmi.12006 [doi].

8. Martinez B, Zomer AL, Rodriguez A, Kok J, Kuipers OP. Cell envelope stress induced by the bacteriocin Lcn972 is sensed by the Lactococcal two-component system CesSR. Mol Microbiol. 2007 Apr; 64(2):473-86. MMI5668 [pii];10.1111/j.1365-2958.2007.05668.x [doi].

9. Schnorpfeil A, Kranz M, Kovacs M, Kirsch C, Gartmann J, Brunner I et al. Target evaluation of the non-coding csRNAs reveals a link of the two-component regulatory system CiaRH to competence control in *Streptococcus pneumoniae* R6. Mol Microbiol. 2013 Jul; 89(2):334-49. 10.1111/mmi.12277 [doi].

10. Campelo AB, Lopez-Gonzalez MJ, Escobedo S, Janzen T, Neves AR, Rodriguez A et al. Mutations Selected After Exposure to Bacteriocin Lcn972 Activate a Bce-Like Bacitracin Resistance Module in *Lactococcus lactis*. Front Microbiol. 2020; 111805. 10.3389/fmicb.2020.01805 [doi].

11. Tymoszewska A, Ovchinnikov KV, Diep DB, Slodownik M, Maron E, Martinez B et al. *Lactococcus lactis* Resistance to Aureocin A53- and Enterocin L50-Like Bacteriocins and Membrane-Targeting Peptide Antibiotics Relies on the YsaCB-KinG-LlrG Four-Component System. Antimicrob Agents Chemother. 2021 Nov 17; 65(12):e0092121. 00921-21 [pii];aac.00921-21 [pii];10.1128/AAC.00921-21 [doi].

12. Silberberg JM, Ketter S, Bohm PJN, Jordan K, Wittenberg M, Grass J et al. KdpD is a tandem serine histidine kinase that controls K(+) pump KdpFABC transcriptionally and post-translationally. Nat Commun. 2024 Apr 15; 15(1):3223. 10.1038/s41467-024-47526-8 [pii];47526 [pii];10.1038/s41467-024-47526-8 [doi].

13. Laroute V, Aubry N, Audonnet M, Mercier-Bonin M, Daveran-Mingot ML, Cocaign-Bousquet M. Natural diversity of lactococci in gamma-aminobutyric acid (GABA) production and genetic and phenotypic determinants. Microb Cell Fact. 2023 Sep 9; 22(1):178. 10.1186/s12934-023-02181-4 [pii];2181 [pii];10.1186/s12934-023-02181-4 [doi].

14. Sanders JW, Leenhouts K, Burghoorn J, Brands JR, Venema G, Kok J. A chloride-inducible acid resistance mechanism in *Lactococcus lactis* and its regulation. Mol Microbiol. 1998 Jan; 27(2):299-310. 10.1046/j.1365-2958.1998.00676.x [doi].
