# Supplementary material for: Unveiling the regulatory network controlling natural transformation in lactococci
Source: PLoS Genet. 2024 Jul 1;20(7):e1011340. doi: 10.1371/journal.pgen.1011340 (PMC11244767; doi:10.1371/journal.pgen.1011340)
Supplement: S4 Table — (PDF) [file pgen.1011340.s014.pdf]

S4 Table. Strains used and generated in this study

Species/Strain	Characteristics	Source
E. coli		
Top 10	F⁻ *mcrA* Δ(*mrr hsdRMS-mcrBC*) φ80*lac*ZΔM15 Δ*lacX74 recA1 deoR ara*D139 Δ(*ara-leu*)7697 *galU galK rpsL endA1 nupG*	Invitrogen
L. lactis		
1AA59	Wild-type dairy isolate	[1]
1526	Wild-type dairy isolate	IFF/Danisco collection
DGCC1594	Wild-type dairy isolate	IFF/Danisco collection
DGCC12650	Wild-type plant isolate	IFF/Danisco collection
DGCC12651	Wild-type plant isolate	IFF/Danisco collection
DGCC12653	Wild-type plant isolate	IFF/Danisco collection
DGCC12657	Wild-type plant isolate	IFF/Danisco collection
DGCC12658	Wild-type plant isolate	IFF/Danisco collection
DGCC12660	Wild-type plant isolate	IFF/Danisco collection
DGCC12662	Wild-type plant isolate	IFF/Danisco collection
DGCC12668	Wild-type plant isolate	IFF/Danisco collection
DGCC12669	Wild-type plant isolate	IFF/Danisco collection
DGCC12671	Wild-type plant isolate	IFF/Danisco collection
DGCC12675	Wild-type plant isolate	IFF/Danisco collection
DGCC12678	Wild-type plant isolate	IFF/Danisco collection
DGCC12685	Wild-type plant isolate	IFF/Danisco collection
DGCC12686	Wild-type plant isolate	IFF/Danisco collection
DGCC12691	Wild-type plant isolate	IFF/Danisco collection
DGCC12696	Wild-type plant isolate	IFF/Danisco collection
FRT101	DGCC12653 *codY*::P_{32}-*cat*	This study
FRT102	DGCC12653 *covRS*::P_{32}-*cat* (*01910-01915*)	This study
FRT103	DGCC12653 *mecA*::P_{32}-*cat*	This study
FRT104	DGCC12653 *ccpA*::P_{32}-*cat*	This study
FRT105	DGCC12653 *covRS*::P_{32}-*cat codY*::*spec*	This study
FRT106	DGCC12653 *comX*::P_{32}-*cat*	This study
FRT107	DGCC12653 *comEC*::P_{32}-*cat*	This study
FRT108	DGCC12653 *mecA* (L-125)	This study
FRT109	DGCC12671 *mecA* (R-125)	This study
FRT201	DGCC1594 *mecA*::P_{32}-*cat*	This study
FRT202	DGCC12650 *mecA*::P_{32}-*cat*	This study
FRT203	DGCC12651 *mecA*::P_{32}-*cat*	This study
FRT204	DGCC12657 *mecA*::P_{32}-*cat*	This study
FRT205	DGCC12660 *mecA*::P_{32}-*cat*	This study
FRT206	DGCC12669 *mecA*::P_{32}-*cat*	This study
FRT207	DGCC12671 *mecA*::P_{32}-*cat*	This study
FRT208	DGCC12675 *mecA*::P_{32}-*cat*	This study
FRT209	DGCC12678 *mecA*::P_{32}-*cat*	This study
FRT210	DGCC12685 *mecA*::P_{32}-*cat*	This study
FRT211	DGCC12691 *mecA*::P_{32}-*cat*	This study
FRT211	DGCC12651 *codY*::P_{32}-*cat*	This study
FRT212	DGCC12651 *covRS*::P_{32}-*cat*	This study
FRT213	DGCC12671 *codY*::P_{32}-*cat*	This study
FRT214	DGCC12671 *covRS*::P_{32}-*cat*	This study
FRT215	DGCC12678 *codY*::P_{32}-*cat*	This study
FRT216	DGCC12678 *covRS*::P_{32}-*cat*	This study
FRT217	DGCC12653 *ecto::nisRK-spc*	This study
FRT218	DGCC12653 *00250*::*spc*	This study
FRT219	DGCC12653 *03130*::*spc*	This study
FRT220	DGCC12653 *04225*::*spc*	This study
FRT221	DGCC12653 *05990*::*spc*	This study
FRT222	DGCC12653 *07535*::*spc*	This study
FRT223	DGCC12653 *08355*::*spc*	This study
FRT224	DGCC12653 *09325*::*spc*	This study
FRT225	DGCC12653 *11640*::*spc*	This study
FRT226	DGCC12653 *12925*::*spc*	This study
FRT227	DGCC12653 *13335*::*spc*	This study
FRT228	DGCC12653 *01140-01145*::P_{32}-*cat*	This study
FRT229	DGCC12653 *03165-03175*::P_{32}-*cat*	This study
FRT230	DGCC12653 *03735-03740*::P_{32}-*cat*	This study
FRT231	DGCC12653 *04255-04260*::P_{32}-*cat*	This study

FRT232	DGCC12653 *04650-04655*::P_{32}-*cat*	This study
FRT233	DGCC12653 *02295-02300*::P_{32}-*cat*	This study

Reference

1. Ladero V, Del RB, Linares DM, Fernandez M, Mayo B, Martin MC et al. Draft Genome Sequence of the Putrescine-Producing Strain *Lactococcus lactis* subsp. *lactis* 1AA59. Genome Announc. 2015 Jun 18; 3(3). 3/3/e00669-15 [pii];genomeA00669-15 [pii];10.1128/genomeA.00669-15 [doi].
