# Supplementary material for: Unveiling the regulatory network controlling natural transformation in lactococci
Source: PLoS Genet. 2024 Jul 1;20(7):e1011340. doi: 10.1371/journal.pgen.1011340 (PMC11244767; doi:10.1371/journal.pgen.1011340)
Supplement: S5 Table — (PDF) [file pgen.1011340.s015.pdf]

S5 Table. Plasmids used and generated in this study

Plasmid	Characteristics	Source or reference
pBAD/His	Ap^r; expression vector	Invitrogen
pBAD_6his-*ccpA*	Ap^r; pBAD derivative carrying 6his-*ccpA* under the control of the arabinose-inducible promoter P_{araB}	This study
pG^+host9	Em^r Ts	[1]
pNZ5319	Em^r Cm^r; pACYC184 derivative containing the P_{32}-*cat* cassette surrounded by *lox* sites	[2]
pJUD-*spc*	Em^r Spc^r; pG^+host9 derivative carrying the *spc* cassette	L. Fontaine (laboratory collection)
pJIM4900	Em^r Ts; pG^+host9 derivative containing the *luxAB* genes of *Photorhabdus luminescens*	E. Guédon (laboratory collection)
pGhP_{xylT}-*comX*	Em^r Ts; pG^+host9 derivative carrying *comX* under the control of the inducible promoter P_{xylT} both cloned from *L. lactis* IO-1	[3]
pGhP_{comX}-*luxAB*	Em^r Ts; pG^+host9 derivative carrying *luxAB* genes under the control of the early promoter P_{comX} of *L. lactis* DGCC12653	This study
pGhP_{comGA}-*luxAB*	Em^r Ts; pG^+host9 derivative carrying *luxAB* genes under the control of the late promoter P_{comGA} of *L. lactis* IO-1	This study
pGhP_{comX}-gfp^{sf}	Em^r Ts; pG^+host9 derivative carrying gfp^{sf} genes under the control of the early promoter P_{comX} of *L. lactis* DGCC12653	This study
pDR111_gfp^{sf}(*Bs*)	*bla amyE'* $P_{hyperspank}$-*sfgfp*(*Bs*) *spc lacI 'amyE*	[4]
pGEM-*rpsL**	Ap^r; pGEM-T Easy derivative carrying the *rpsL** gene from *L. lactis* IL1403	This study
pNZ8048	Cm^r; Translational fusion vector carrying P_{nisA} + terminator	[5]
pNZ8048_6his-*codY*	Cm^r; pNZ8048 derivative carrying 6his-*codY* under the control of the nisin-inducible promoter P_{nisA}	This study
pNZ8048_6his-*covR*	Cm^r; pNZ8048 derivative carrying 6his-*covR* under the control of the nisin-inducible promoter P_{nisA}	This study

Ap^r, Em^r, Cm^r, and Spc^r: ampicillin, erythromycin, chloramphenicol, and spectinomycin resistance, respectively
Ts: thermosensitive

References

1. Maguin E, Prevost H, Ehrlich SD, Gruss A. Efficient insertional mutagenesis in lactococci and other gram-positive bacteria. J Bacteriol. 1996 Feb; 178(3):931-5. 10.1128/jb.178.3.931-935.1996 [doi].

2. Lambert JM, Bongers RS, Kleerebezem M. Cre-lox-based system for multiple gene deletions and selectable-marker removal in Lactobacillus plantarum. Appl Environ Microbiol. 2007 Feb; 73(4):1126-35. AEM.01473-06 [pii];1473-06 [pii];10.1128/AEM.01473-06 [doi].

3. David B, Radziejwoski A, Toussaint F, Fontaine L, de Frahan MH, Patout C et al. Natural DNA Transformation Is Functional in *Lactococcus lactis* subsp. *cremoris* KW2. Appl Environ Microbiol. 2017 Aug 15; 83(16). AEM.01074-17 [pii];01074-17 [pii];10.1128/AEM.01074-17 [doi].

4. Overkamp W, Beilharz K, Detert Oude WR, Solopova A, Karsens H, Kovacs A et al. Benchmarking various green fluorescent protein variants in *Bacillus subtilis*, *Streptococcus pneumoniae*, and *Lactococcus lactis* for live cell imaging. Appl Environ Microbiol. 2013 Oct; 79(20):6481-90. AEM.02033-13 [pii];02033-13 [pii];10.1128/AEM.02033-13 [doi].

5. Kuipers OP, Ruyter PGGAD, Kleerebezem M, de Vos WMD. Quorum sensing-controlled gene expression in lactic acid bacteria. J Biotechnol. 1998; 64(1):15-21.
