# Supplementary material for: Unveiling the regulatory network controlling natural transformation in lactococci
Source: PLoS Genet. 2024 Jul 1;20(7):e1011340. doi: 10.1371/journal.pgen.1011340 (PMC11244767; doi:10.1371/journal.pgen.1011340)
Supplement: S6 Table — (PDF) [file pgen.1011340.s016.pdf]

S6 Table. Oligonucleotides used in this study

Target fragment	Primer name	Primer sequence (5'-3')	Template DNA	PCR fragment size
codY::P32-*cat*				
Up recombination arm	FT767_codY_locus_fw	CTTTTGGACACAGGGGATGAGG	DGCC12653 chromosome	1148 bp
	FT768_codY_rec_rv	GCCCTTATGGGATTTATCTTCCTTATCAGTCATACTGT TATATGGCAACTCTTGT		
Resistance cassette	252-Uplox66	TAAGGAAGATAAATCCCATA	pNZ5319	1402 bp
	253-DNlox71	TTCACGTTACTAAAGGGAATGTA		
Down recombination arm	FT769_codY_rec_fw	TCTACATTCCCTTTAGTAACGTGAAACTGGTTTATTTG ATAAACTTGCAGGACGT	DGCC12653 chromosome	1225 bp
	FT770_codY_locus_rv	GTTATTGTATGACATTAAGCATC		
Insertion validation	252-Uplox66	TAAGGAAGATAAATCCCATA	clones	2800 bp
	FT771_codY_diagR	TCCGTCAGTCACAACGTCTGG		
covRS::P32-*cat* (*01910-01915*::P32-*cat*)				
Up recombination arm	FT732_01915_locus_fw	CAACTTTGGTCTGGCAAACAGTCC	DGCC12653 chromosome	1223 bp
	FT733_019115_rec_rv	GCCCTTATGGGATTTATCTTCCTTAGTCGCATAGCCTT CATGTTCTAATTCTAAT		
Resistance cassette	252-Uplox66	TAAGGAAGATAAATCCCATA	pNZ5319	1402 bp
	253-DNlox71	TTCACGTTACTAAAGGGAATGTA		
Down recombination arm	FT734_01915_rec_fw	TCTACATTCCCTTTAGTAACGTGAATTTAGCTCGATTG GCAGAAAATTATCAAGG	DGCC12653 chromosome	1272 bp
	FT735_01915_locus_rv	ATCCCGCCAATACCGTGACAAC		
Insertion validation	252-Uplox66	TAAGGAAGATAAATCCCATA	clones	2805 bp
	FT736_01915_diagR	ATGACCCAACTCACAATAGC		
mecA::P32-*cat*				
Up recombination arm	FT681_mecA_locus_fw	TTTAATCCTTGCAAAGAAGCAACCGAGA	DGCC12653 chromosome	1104 bp
	FT 679_mecA_rec_fw	CGCCCTTATGGGATTTATCTTCCTTACGTTATGAAGAT ATTATGTCTGTCTTAAC		
Resistance cassette	252-Uplox66	TAAGGAAGATAAATCCCATA	pNZ5319	1402 bp
	253-DNlox71	TTCACGTTACTAAAGGGAATGTA		
Down recombination arm	FT680_mecA_rec_fw	TCTACATTCCCTTTAGTAACGTGAATATCACTTTACCA GTCTTATTAGTATTATA	DGCC12653 chromosome	1100 bp
	FT682_mecA_locus_rv	CTTTGAGAAACATTCTACCAGCCGTC		
Insertion validation	252-Uplox66	TAAGGAAGATAAATCCCATA	clones	2713 bp
	FT683_mecA_diagR	TCTCATATTATAAAAGCCAGTCATTAG		
ccpA::P32-*cat*				
Up recombination arm	FT720_ccpA_locus_fw	CGTACCCCTATTTAGAATACGC	DGCC12653 chromosome	1226 bp
	FT719_ccpA_DGCC_rec_rv	GCCCTTATGGGATTTATCTTCCTTAAGAAGATGTTGAA CAAAATCAATTAGTTTTGG		
Resistance cassette	252-Uplox66	TAAGGAAGATAAATCCCATA	pNZ5319	1402 bp
	253-DNlox71	TTCACGTTACTAAAGGGAATGTA		
Down recombination arm	FT634_CcpA_kw2_rec_fw	CTACATTCCCTTTAGTAACGTGAACATAGTATTTATGA AAACCATTTTCATTTAC	DGCC12653 chromosome	1276 bp
	FT635_CcpA_kw2_locus_rv	GGTCAGTATGAGTGAAACTTTCACAAC		
Insertion validation	252-Uplox66	TAAGGAAGATAAATCCCATA	clones	2941 bp
	FT721_ccpA_diagR	ATGCACCAGATGGTCGGTA		
codY::*spc*				

Up recombination arm	FT767_codY_locus_fw	CTTTTGGACACAGGGGATGAGG	DGCC12653 chromosome	1148 bp
	FT768_codY_rec_rv	GCCCTTATGGGATTTATCTTCCTTATCAGTCATACTGTTATATGGCAACTCTTGT		
Resistance cassette	252-Uplox66	TAAGGAAGATAAATCCCATA	pJUD-*spc*	1034 bp
	253-DNlox71	TTCACGTTACTAAAGGGAATGTA		
Down recombination arm	FT769_codY_rec_fw	TCTACATTCCCTTTAGTAACGTGAAACTGGTTTATTTGATAAACTTGCAGGACGT	DGCC12653 chromosome	1225 bp
	FT770_codY_locus_rv	GTTATTGTATGACATTAAGCATC		
Insertion validation	252-Uplox66	TAAGGAAGATAAATCCCATA	clones	2432 bp
	FT771_codY_diagR	TCCGTCAGTCACAACGTCTGG		
ecto::*nisRK-spc*				
Up recombination arm	FT929_ecto_1500_fw	CTTGCTTCTTGATAAAGGAAAGTTGCA	DGCC12653 chromosome	1527 bp
	FT1125_nisRK_ecto_rec_rv	TGATATTAAATCTGGAACAGTCTGTGGGCCGCCCAATATGACAAGAGCGACTAT		
nisRK genes	FT1055_nisRK_aval	CCACAGACTGTTCCAGATTTAATATCA	IO-1 chromosome	2665 bp
	FT1057_nisRK_spec_rec	ATCCTTATGGGATTTATCTTCCTTAAAGCTTTTAGCTTAGATACAGATAAAG		
Resistance cassette	252-Uplox66	TAAGGAAGATAAATCCCATA	pJUD-*spc*	1034 bp
	253-DNlox71	TTCACGTTACTAAAGGGAATGTA		
Down recombination arm	FT711_ectopic_rec_fw	ATCTACATTCCCTTTAGTAACGTGAAATACCAGTTTGACTTTACCAAAGTATAGTG	DGCC12653 chromosome	1523 bp
	FT934_ecto_1500_rv	AAATTTGTAAGCAATCGCCCAGCGCG		
Insertion validation	FT930_ecto_1750_fw	AATTGCGTTAAATAATATCATTCA	clones	5424 bp
	253-DNlox71	TTCACGTTACTAAAGGGAATGTA		
comX::P_{32}-*cat*				
Up recombination arm	FT694_comX_locus_fw	TGACCATGTTACACAAGCCTATATCCT	DGCC12653 chromosome	1254 bp
	FT_693_comXcat_rec_rv	CGCCCTTATGGGATTTATCTTCCTTACTTCGTTTCTTTGCATAACTTCGTCTTAAT		
Resistance cassette	252-Uplox66	TAAGGAAGATAAATCCCATA	pNZ5319	1402 bp
	253-DNlox71	TTCACGTTACTAAAGGGAATGTA		
Down recombination arm	FT692_comXcat_rec_fw	TCTACATTCCCTTTAGTAACGTGAACCATGACCATTTTATAGGTTTAGATGTTTATG	DGCC12653 chromosome	1505 bp
	288_AR_ComX_DN_luxR	CCCAACATCTCACGACAC		
Insertion validation	252-Uplox66	TAAGGAAGATAAATCCCATA	clones	3343 bp
	CP_comXRVdiag	ATTCCTTAGAAAGGAGGTGATC		
comEC::P_{32}-*cat*				
Up recombination arm	FT685_comEC_DGCC_locus_fw	GTGAAGATCAGCCAACCACTCTTTCCA	DGCC12653 chromosome	974 bp
	FT684_comEC_DGCC_rec_rv	CGCCCTTATGGGATTTATCTTCCTTAGTAAAGGCAATAAGATTACATCAAATAA		
Resistance cassette	252-Uplox66	TAAGGAAGATAAATCCCATA	pNZ5319	1402 bp
	253-DNlox71	TTCACGTTACTAAAGGGAATGTA		
Down recombination arm	FT 686 comEC DGCC rec fw	ATCTACATTCCCTTTAGTAACGTGAATATTAGGAACTTTTCTCTTGTCTCTAATTGG	DGCC12653 chromosome	1227 bp
	FT 687 comEC DGCC locus rv	TCAAAGTGTGCGCTGTGAAGTCATTACTC		
Insertion validation	252-Uplox66	TAAGGAAGATAAATCCCATA	clones	3445 bp
	BlD_diagINTcomECRV1	GTCCAATAATACCATTCATGAAC		
DGCC12653_00250::*spc*				
Up recombination arm	FT772_00250_locus_fw	ATTGATGAAGTTGGTCGTGGA	DGCC12653 chromosome	1284 bp
	FT773_00250_rec_rv	ATCCTTATGGGATTTATCTTCCTTACATCAAATCTATGCGCTCAAAACTCATCAT		
Resistance cassette	252-Uplox66	TAAGGAAGATAAATCCCATA	pJUD-*spc*	1034 bp

	253-DNlox71	TTCACGTTACTAAAGGGAATGTA		
Down recombination arm	FT778_00250_rec_fw	ATTACATTCCCTTTAGTAACGTGAATAGTCTCTTTTGA GCTGTTGCTT	DGCC12653 chromosome	1496 bp
	FT779_00250_locus_rv	GTTATTGTATGACATTAAGCATC		
Insertion validation	252-Uplox66	TAAGGAAGATAAATCCCATA	clones	2505 bp
	FT780_00250_diagR	TCCGTCAGTCACAACGTCTGG		
DGCC12653_03130::*spc*				
Up recombination arm	FT781_03130_locus_fw	GGAGCGTTTAGACGCACCAACA	DGCC12653 chromosome	1216 bp
	FT782_03130_rec_rv	ATCCTTATGGGATTTATCTTCCTTAACCATTCCACATT CCAGCATTTCAATTTA		
Resistance cassette	252-Uplox66	TAAGGAAGATAAATCCCATA	pJUD-*spc*	1034 bp
	253-DNlox71	TTCACGTTACTAAAGGGAATGTA		
Down recombination arm	FT783_03130_rec_fw	ATTACATTCCCTTTAGTAACGTGAAATTGGGTTTGCCA GAGATGAAGGCTTATT	DGCC12653 chromosome	1267 bp
	FT784_03130_locus_rv	ATTGCTTCTGGTATGATAATAAG		
Insertion validation	252-Uplox66	TAAGGAAGATAAATCCCATA	clones	2449 bp
	FT785_03130_diagR	TCAGCCGAATAGTTTGTATAAAC		
DGCC12653_04225::*spc*				
Up recombination arm	FT786_04225_locus_fw	GGTTTGCGATAATCATTCTTGTA	DGCC12653 chromosome	1151 bp
	FT787_04225_rec_rv	ATCCTTATGGGATTTATCTTCCTTAGGTTTGACCTAAT TCAAATCTTGATAAAC		
Resistance cassette	252-Uplox66	TAAGGAAGATAAATCCCATA	pJUD-*spc*	1034 bp
	253-DNlox71	TTCACGTTACTAAAGGGAATGTA		
Down recombination arm	FT788_04225_rec_fw	ATTACATTCCCTTTAGTAACGTGAATAGGAAAATTATT AACATAAAAAAGTCTC	DGCC12653 chromosome	1265 bp
	FT789_04225_locus_rv	CATTACTACTTCCCTACTAACGC		
Insertion validation	252-Uplox66	TAAGGAAGATAAATCCCATA	clones	2436 bp
	FT790_04225_diagR	TAAGGAAGATAAATCCCATA		
DGCC12653_05990::*spc*				
Up recombination arm	FT 791 05990 locus fw	TACGGTGATTCCTGACCGTATCG	DGCC12653 chromosome	1025 bp
	FT 792 05990 rec rv	ATCCTTATGGGATTTATCTTCCTTATAACTACGGTCTA TTTGAAAGCATTATATG		
Resistance cassette	252-Uplox66	TAAGGAAGATAAATCCCATA	pJUD-*spc*	1034 bp
	253-DNlox71	TTCACGTTACTAAAGGGAATGTA		
Down recombination arm	FT 793 05990 rec fw	ATTACATTCCCTTTAGTAACGTGAATGTTCATATATGA TTATTTTTGTTTATTA	DGCC12653 chromosome	1247 bp
	FT 794 05990 locus rv	CACTCCTAACTGTTTCCACATC		
Insertion validation	252-Uplox66	TAAGGAAGATAAATCCCATA	clones	2464 bp
	FT 795 05990 diagR	GCTTCTGGTCGCCTTGTTACC		
DGCC12653_07535::*spc*				
Up recombination arm	FT796_07535_locus_fw	ATACAGGTGGGATTGAACTTTCA	DGCC12653 chromosome	1281 bp
	FT797_07535_rec_rv	ATCCTTATGGGATTTATCTTCCTTAATATACCATTTAA TCTTCTTTCAATTAAC		
Resistance cassette	252-Uplox66	TAAGGAAGATAAATCCCATA	pJUD-*spc*	1034 bp
	253-DNlox71	TTCACGTTACTAAAGGGAATGTA		
Down recombination arm	FT798_07535_rec_fw	ATTACATTCCCTTTAGTAACGTGAAGTATGATAAAAG AATACTAAGAGAA	DGCC12653 chromosome	1228 bp
	FT799_07535_locus_rv	CTATCCGTATTGCGAATTGC		
Insertion validation	252-Uplox66	TAAGGAAGATAAATCCCATA	clones	2351 bp
	FT800_07535_diagR	TAGCTTGTGTCGGTTGCAAAG		

DGCC12653_08355::*spc*				
Up recombination arm	FT801_08355_locus_fw	CGCAAGTACCCTTTATGGTGGA	DGCC12653 chromosome	1258 bp
	FT802_08355_rec_rv	ATCCTTATGGGATTTATCTTCCTTAGCTTCTCCTTGATAAGTAAAATATTTAACA		
Resistance cassette	252-Uplox66	TAAGGAAGATAAATCCCATA	pJUD-*spc*	1034 bp
	253-DNlox71	TTCACGTTACTAAAGGGAATGTA		
Down recombination arm	FT803_08355_rec_fw	ATTACATTCCCTTTAGTAACGTGAACCGTAGTGATAACACTACGGCTTTTTTGT	DGCC12653 chromosome	1227 bp
	FT804_08355_locus_rv	GCCCTTATTTTATCATAAATCTTGG		
Insertion validation	252-Uplox66	TAAGGAAGATAAATCCCATA	clones	2585 bp
	FT805_08355_diagR	ATCATCACGACATCAAGTGG		
DGCC12653_09325::*spc*				
Up recombination arm	FT806_09325_locus_fw	CATGGTTAGAACTCAATAACTATG	DGCC12653 chromosome	1224 bp
	FT807_09325_rec_rv	ATCCTTATGGGATTTATCTTCCTTATTGTTTCATCTAATTATCCTTCTTTTA		
Resistance cassette	252-Uplox66	TAAGGAAGATAAATCCCATA	pJUD-*spc*	1034 bp
	253-DNlox71	TTCACGTTACTAAAGGGAATGTA		
Down recombination arm	FT808_09325_rec_fw	ATTACATTCCCTTTAGTAACGTGAACGCCATGTTCTTAATTTAGAAGAATCA	DGCC12653 chromosome	1228 bp
	FT809_09325_locus_rv	ACGTCCACCACCACATACAGTC		
Insertion validation	252-Uplox66	TAAGGAAGATAAATCCCATA	clones	2385 bp
	FT_810_09325_diagR	CCATTACAGCAATATAAACATCCA		
DGCC12653_11640::*spc*				
Up recombination arm	FT811_11640_locus_fw	GACAGCTTCTCATAAGTCATC	DGCC12653 chromosome	1239 bp
	FT812_11640_rec_rv	ATCCTTATGGGATTTATCTTCCTTACCATAGTATTGATTAACCATTTGTCTTGTC		
Resistance cassette	252-Uplox66	TAAGGAAGATAAATCCCATA	pJUD-*spc*	1034 bp
	253-DNlox71	TTCACGTTACTAAAGGGAATGTA		
Down recombination arm	FT813_11640_rec_fw	ATTACATTCCCTTTAGTAACGTGAAATCGGTGCTACAATAAAGTTAAGGAGACA	DGCC12653 chromosome	1278 bp
	FT814_11640_locus_rv	CCAAGCAAAGGTCGCTAAAGTTC		
Insertion validation	252-Uplox66	TAAGGAAGATAAATCCCATA	clones	2380 bp
	FT815_11640_diagR	ATCCGAATCACCAAGATAGGCG		
DGCC12653_12925::*spc*				
Up recombination arm	FT821_12925_locus_fw	GCAGTTGCCATTATTCCTGGTATC	DGCC12653 chromosome	1305 bp
	FT822_12925_rec_rv	ATCCTTATGGGATTTATCTTCCTTATTTGAGTCCATAAATTATCCTCATT		
Resistance cassette	252-Uplox66	TAAGGAAGATAAATCCCATA	pJUD-*spc*	1034 bp
	253-DNlox71	TTCACGTTACTAAAGGGAATGTA		
Down recombination arm	FT823_12925_rec_fw	ATTACATTCCCTTTAGTAACGTGAATTAGGTTATGACAAAGCGGCAAGTTATATG	DGCC12653 chromosome	1299 bp
	FT824_12925_locus_rv	GAAGCATAGCATAAATTGGAAGCG		
Insertion validation	252-Uplox66	TAAGGAAGATAAATCCCATA	clones	2367 bp
	FT825_12925_diagR	CTAATCCATCTGACCCTATGACAT		
DGCC12653_13335::*spc*				
Up recombination arm	FT831_13335_locus_fw	5'TCTCGATTGGTCTAATTGTTGTTG3'	DGCC12653 chromosome	1130 bp
	FT832_13335_rec_rv	5'ATCCTTATGGGATTTATCTTCCTTATTCATTTTCCCTTTGTCATATATTCCA3'		
Resistance cassette	252-Uplox66	TAAGGAAGATAAATCCCATA	pJUD-*spc*	1034 bp
	253-DNlox71	TTCACGTTACTAAAGGGAATGTA		

Down recombination arm	FT833_13335_rec_fw	ATTACATTCCCTTTAGTAACGTGAAAATTATAGGGCTTTGGCATATATTCCA	DGCC12653 chromosome	1229 bp
	FT834_13335_locus_rv	CTGAACTAACAACGAAATTAACAAAC		
Insertion validation	252-Uplox66	TAAGGAAGATAAATCCCATA	clones	2317 bp
	FT835_13335_diagR	GACACACAATTTCCGCACCAT		
DGCC12653_01140-DGCC12653_01145::P_{32}- *cat*				
Up recombination arm	FT722_01140_locus_fw	AGTCGAACCCCTGTCCAAACAC	DGCC12653 chromosome	1171 bp
	FT723_01140_rec_rv	GCCCTTATGGGATTTATCTTCCTTAAGTTACTTCGTAATCATTTTGCCGAAAATT		
Resistance cassette	252-Uplox66	TAAGGAAGATAAATCCCATA	pNZ5319	1402 bp
	253-DNlox71	TTCACGTTACTAAAGGGAATGTA		
Down recombination arm	FT724_01140_rec_fw	ATCTACATTCCCTTTAGTAACGTGAAAGGAACAACTTTCACCGTCAATCTTTAATCA	DGCC12653 chromosome	1185 bp
	FT725_01140_locus_rv	CTGTCAGTCGAATGACAGCA		
Insertion validation	252-Uplox66	TAAGGAAGATAAATCCCATA	clones	2593 bp
	FT726_01140_diagR	TCCAAGCGAACGTGATTTGGC		
DGCC12653_02295-DGCC12653_02300::P_{32}-*cat*				
Up recombination arm	FT762_02295_locus_fw	ATGGTGAGCAAGAACGTCAGGT	DGCC12653 chromosome	1110 bp
	FT763_02295_rec_rv	GCCCTTATGGGATTTATCTTCCTTATTCTTGTCCATATTATAGCATAAAAAAACGT		
Resistance cassette	252-Uplox66	TAAGGAAGATAAATCCCATA	pNZ5319	1402 bp
	253-DNlox71	TTCACGTTACTAAAGGGAATGTA		
Down recombination arm	FT764_02295_rec_fw	TCTACATTCCCTTTAGTAACGTGAAGAAGATTGAACTTGATTCCAATTATCCA	DGCC12653 chromosome	1260 bp
	FT765_02295_locus_rv	CTGAAACCATATATCCAAACGC		
Insertion validation	252-Uplox66	TAAGGAAGATAAATCCCATA	clones	2839 bp
	FT766_02295_diagR	CCTTCCATGTTACCCATAGCTTGAC		
DGCC12653_03165-DGCC12653_03175::P_{32}-*cat*				
Up recombination arm	FT695_03175_locus_fw	GTAGAGTTGTCTATGTTAATGATG	DGCC12653 chromosome	1402 bp
	FT696_03175_rec_rv	CGCCCTTATGGGATTTATCTTCCTTAAATCAACTTTTAAGTAATCAAGTTCATAGA		
Resistance cassette	252-Uplox66	TAAGGAAGATAAATCCCATA	pNZ5319	1402 bp
	253-DNlox71	TTCACGTTACTAAAGGGAATGTA		
Down recombination arm	FT697_03175_rec_fw	TCTACATTCCCTTTAGTAACGTGAATACAACAGAAGTGGCTGGTAAAACAGTCAA	DGCC12653 chromosome	1452 bp
	FT698_03175_locus_rv	ACGATTGAAACTTTGACTTAACTGATG		
Insertion validation	252-Uplox66	TAAGGAAGATAAATCCCATA	clones	2900 bp
	FT699_03175_diagR	CTTAGCTCATGTGAAGCATCCGAG		
DGCC12653_03735-DGCC12653_03740::P_{32}-*cat*				
Up recombination arm	FT737_03735_locus_fw	GAGATACGTTAGCCAATGGGA	DGCC12653 chromosome	1233 bp
	FT738_03735_rec_rv	GCCCTTATGGGATTTATCTTCCTTAAACGAGTCGCCAAGATAGTAAAGAGTATAG		
Resistance cassette	252-Uplox66	TAAGGAAGATAAATCCCATA	pNZ5319	1402 bp
	253-DNlox71	TTCACGTTACTAAAGGGAATGTA		
Down recombination arm	FT739_03735_rec_fw	TCTACATTCCCTTTAGTAACGTGAAGCAACAATTTATGCCATTCAACATCACTTA	DGCC12653 chromosome	1284 bp
	FT740_03735_locus_rv	CTCCGTAGATTGAAGAACCT		
Insertion validation	252-Uplox66	TAAGGAAGATAAATCCCATA	clones	2846 bp
	FT741_03735_diagR	GCCAGTATATGTTTCAGCTAC		
DGCC12653_04255-DGCC12653_04260::P_{32}-*cat*				

Up recombination arm	FT742_04260_locus_fw	GTGAAAATATGGCAGTTACAA	DGCC12653 chromosome	1225 bp
	FT743_04260_rec_rv	GCCCTTATGGGATTTATCTTCCTTAAGAGTGATAAATCATCCTCCACTAATAA		
Resistance cassette	252-Uplox66	TAAGGAAGATAAATCCCATA	pNZ5319	1402 bp
	253-DNlox71	TTCACGTTACTAAAGGGAATGTA		
Down recombination arm	FT744_04260_rec_fw	TCTACATTCCCTTTAGTAACGTGAAGTGTGGATAGCACAATTTCAAATCCTGCA	DGCC12653 chromosome	1302 bp
	FT745_04260_locus_rv	ATCATAAGCACGTTGAACAA		
Insertion validation	252-Uplox66	TAAGGAAGATAAATCCCATA	clones	2896 bp
	FT746_04260_diagR	GAATCACAACTGGTTCTTCAT		
DGCC12653_04650-DGCC12653_04655::P_{32}-*cat*				
Up recombination arm	FT747_04655_locus_fw	CTCGGCACTAAATGTATCTT	DGCC12653 chromosome	1198 bp
	FT748_04655_rec_rv	GCCCTTATGGGATTTATCTTCCTTAGCTTTCACAATAATTTCATCATCTTCTAC		
Resistance cassette	252-Uplox66	TAAGGAAGATAAATCCCATA	pNZ5319	1402 bp
	253-DNlox71	TTCACGTTACTAAAGGGAATGTA		
Down recombination arm	FT749_04655_rec_fw	TCTACATTCCCTTTAGTAACGTGAATGAACATGGATTTACTGGATATAATGGTC	DGCC12653 chromosome	1282 bp
	FT750_04655_locus_rv	TTAAGACTAAGAAAGTCCTATCTT		
Insertion validation	252-Uplox66	TAAGGAAGATAAATCCCATA	clones	2836 bp
	FT751_04655_diagR	GCCAAACATAATACTACGATGT		
DGCC12653_06415-DGCC12653_06420::P_{32}-*cat*				
Up recombination arm	FT752_06420_locus_fw	GGCAACAAGACGACAGGAACGA	DGCC12653 chromosome	1175 bp
	FT753_06420_rec_rv	GCCCTTATGGGATTTATCTTCCTTATCATAGTTGTCCTTTCAAGTGGAA		
Resistance cassette	252-Uplox66	TAAGGAAGATAAATCCCATA	pNZ5319	1402 bp
	253-DNlox71	TTCACGTTACTAAAGGGAATGTA		
Down recombination arm	FT754_06420_rec_fw	TCTACATTCCCTTTAGTAACGTGAAGATGATGGTACTGATGAGTTTGAATTTG	DGCC12653 chromosome	1215 bp
	FT755_06420_locus_rv	CGCGGCTTCTGTTGCTGGTT		
Insertion validation	252-Uplox66	TAAGGAAGATAAATCCCATA	clones	2680 bp
	FT751_04655_diagR	GATTCAATGCTGCAACTGGTCT		
*rpsL** donor DNA				
*rpsL** locus	BlD-LLcfusARpsL	ACACCTTTGTTCTTGAAGG	IL1403 streptomycine^{r}	3712 bp
	BlD-LLldacARpsL	AAGCTGTCAGTAAATTGACG		
*rpsL** sequencing				
*rpsL** gene	BlD-RpsLUnivUp	GTATTTCTCATCGCTTCGC	IL1403 streptomycine^{r}	525 bp
	BlD-RpsLUnivDown	CCAAAACTTCACGTTTTGG		
pGhP$_{comX}$-*luxAB*				
P$_{comX}$	FT886_PcomX_fw	AATAAAAAAAGCAAGGTAAATAGCC	DGCC12653 chromosome	179 bp
	FT887_PcomX_rv	GTATGTAAGCAAAAAGTTTCCAAATTTCATTGGAAAGTTCTCCTTTTATATTATC		
pGh-*luxAB*	FT884_pGhPcomGAlux_fw	ATGAAATTTGGAAACTTTTTGCTTACATAC	pGhP$_{comGA[MG]}$-*luxAB*	5808 bp
	FT925_PcomXlux_rec_rv	AAGGCTATTTACCTTGCTTTTTTATTCTCGAGGGGGGGCCCGGTACCCAATTCGC		
pGhP$_{comGA}$-*luxAB*				
P$_{comGA}$	LuxcIOF1_XhoI	ATAGTCTCGAGAAATAAATGGCTACAAAATT	IO-1 chromosome	479 bp
	LuxIOR1	GTAAGCAAAAAGTTTCCAAATTTCATACTAGACTATACGCAAATAATC		
pGh-*luxAB*	LuxcIOR2_XhoI	ATAGCTCGAGTCCCCTGACGAACTCAAGAAGATGC		5934 bp

	LuxIOF2	GATTATTTGCGTATAGTCTAGTATGAAATTTGGAAACTTTTTGCTTAC	pGhP$_{comGA[MG]}$-*luxAB*	
pGhP$_{comGA}$-*luxAB*				
P$_{comX}$	FT886_PcomX_fw	AATAAAAAAGCAAGGTAAATAGCC	DGCC12653 chromosome	287 bp
	FT968_sfGFP_rec	CACCTGTGAACAGCTCTTCTCCTTTTGACATTGGAAAGTTCTCCTTTTATATTAT		
gfp^{sf} gene	FT967_sfGFP_fw	ATGTCAAAAGGAGAAGAGCTGTTCACAGGT	pDR111-sfGFP(Bs)	737 bp
	FT969_sfGFP_rv_SacII	CTACTGTCCCGCGGTCATTATTACTTATAAAGCTCATCCATGCCGT		
pGh	FT970_pGh_SacII	ATGTCTACCGCGGATCCTCTAGAGTCCGCTAGGG	pG^{+}host9	3721 bp
	FT925_PcomXlux_rec_rv	AAGGCTATTTACCTTGCTTTTTTATTCTCGAGGGGGGGCCCGGTACCCAATTCGC		
pBAD_6his-*ccpA*				
ccpA	FT1447_ccpA_rec_fw	GGCATCACCATCACCATCACGTAGAATCAACAACAACAATTTATGATGTGGCA	DGCC12653 chromosome	1036 bp
	FT1448_ccpA_rec_rv	CCAAAACAGCCAAGCTTCTATTATTTGGTAGAACGACGAGAAAAGATTTCATG		
pBAD-6his	FT1328_pBAD_stop_fw	TAGAAGCTTGGCTGTTTTGGCGGATGAG	pBAD-6his	3985 bp
	FT1446_pBAD6his_rev	GTGATGGTGATGGTGATGCCCCATG		
pNZ8048_6his-*codY*				
6his-*codY*	FT1321_6HiscodY_Up	ATTATAAGGAGGCACTCACCATGGGGCATCACCATCACCATCACGTGGCTACATTACTTGAAAAAAC	DGCC12653 chromosome	853 bp
	FT1322_6HiscodY_Dw	CCAAAACAGCCAAGCTTCTATTATTTGGTAGAACGACGAGAAAAGATTTCATG		
pNZ8048	FT1112_pNZ_Gib_fw	TGAACCAAAATTAGAAAACCAAGGCTTG	pNZ8048	3296 bp
	FT1111_pNZ_Gib_rv	GGTGAGTGCCTCCTTATAATTTATTTTG		
pNZ8048_6his-*covR*				
6his-*covR*	FT1334_pNZ_covR_rec_fw	ATTATAAGGAGGCACTCACCATGGGGCATCACCATCACCATCACACTTCAAAGAAAATTTTGATTATTG	DGCC12653 chromosome	754 bp
	FT1335_pNZ_covR_rec_rv	GGTTTTCTAATTTTGGTTCATTATTTGCGTTCACGCATTACATAACCTAAGC		
pNZ8048	FT1112_pNZ_Gib_fw	TGAACCAAAATTAGAAAACCAAGGCTTG	pNZ8048	3296 bp
	FT1111_pNZ_Gib_rv	GGTGAGTGCCTCCTTATAATTTATTTTG		
mecA locus for gene swapping				
mecA	FT1319_mecA_Up_Up	TGATAAATTCCATAACAGAAACTTTGTCAT	DGCC12653 and DGCC12671 chromosome	5030 bp
	FT1320_mecA_Dw_Dw	GACAAATATTAGACCTTAAAAATCCGGATG		
EMSA: P$_{comX}$				
P$_{comX}$	FT851_Cy3_PcomX_up	Cy3-AATAAAAAAGCAAGGTAAATAGC	DGCC12653 chromosome	256 bp
	FT853_Cy5_PcomX_dw	Cy5-TGGAAAGTTCTCCTTTTATATTATC		
EMSA: X2 Fw				
P$_{comX}$	FT888_PcomX2	GTAATAAAAGCCATAAAAATGTCTTG	DGCC12653 chromosome	154 bp
	FT853_Cy5_PcomX_dw	Cy5-TGGAAAGTTCTCCTTTTATATTATC		
EMSA: X3 Fw				
P$_{comX}$	FT1558	TGTCTTGGAAGGATAGATATGAAAATAGCTCATTATAAT	DGCC12653 chromosome	135 bp
	FT853_Cy5_PcomX_dw	Cy5-TGGAAAGTTCTCCTTTTATATTATC		
EMSA: X4 Fw				
P$_{comX}$	FT889	AAGATGCTCCCTGACTGTTTATTAGAT	DGCC12653 chromosome	96 bp
	FT853_Cy5_PcomX_dw	Cy5-TGGAAAGTTCTCCTTTTATATTATC		
EMSA: X5 Rv				
P$_{comX}$	FT851_Cy3_PcomX_up	Cy3-AATAAAAAAGCAAGGTAAATAGC	DGCC12653 chromosome	86 bp
	FT1355	CGTGTTTTACCTAACGATATTA		

EMSA: X6 Rv				
P_{comX}	FT851_Cy3_PcomX_up	Cy3-AATAAAAAAGCAAGGTAAATAGC	DGCC12653 chromosome	115 bp
	FT975	TGGCTTTTATTACATTATAATAATTTTTACGT		
EMSA: P_{comX} native CodY-box				
P_{comX}	FT1368_PcomX_codY_control	TATTTATTGGGTTATAAATTCTGAATATTCGCATAATATCGTTAGGTAAAAC	DGCC12653 chromosome	225 bp
	FT853_Cy5_PcomX_dw	Cy5-TGGAAAGTTCTCCTTTTATATTATC		
EMSA: P_{comX} mutated CodY-box				
P_{comX}	FT1369_PcomX_codY_mute	TATTTATTGGGTTATAAATTATAAATATTCGCATAATATCGTTAGGTAAAAC	DGCC12653 chromosome	225 bp
	FT853_Cy5_PcomX_dw	Cy5-TGGAAAGTTCTCCTTTTATATTATC		
EMSA: negative control				
CDS *dnaE*	AK350	CCTGTAGTTCCCTTACATAC	*S. salivarius* HSISS4 chromosome	150 bp
	AK303	TTCCATTTCTTGAGGCGAG		
