# Supplementary material for: Unveiling the regulatory network controlling natural transformation in lactococci
Source: PLoS Genet. 2024 Jul 1;20(7):e1011340. doi: 10.1371/journal.pgen.1011340 (PMC11244767; doi:10.1371/journal.pgen.1011340)
Supplement: S9 Fig — The nucleotide sequences correspond to the intergenic regions located upstream of comX (PcomX) in the genomes of the 18 DGCC strains used in this work, the laboratory strain IL1403, and strain KF147 shown to be transformable by comX overexpression. Strains DGCC12653, DGCC12651, DGCC12671 and DGCC12678 in red were selected for codY and covRS inactivation. Red bars indicate putative -10 boxes of vegetative promoters (P1 to P4). CodY-, cre- and CovR-boxes are surrounded in red, green, and blue, respectively. The alignment was performed using Clustal Omega. (PDF) [file pgen.1011340.s009.pdf]

```
PcomX_DGCC1526      --aataaaaaagcaaggtaaatagccttgcttttatttattgggttataaattctgaatattcgcataatatcgttaggtaaaacacgtagaaattattataatgtaataaaagccataaaaatgtcttggaaagatagatat
PcomX_DGCC12658     --aataaaaaagcaaggtaaatagccttgcttttatttattgggttataaattctgaatattcgcataatatcgttcggtaaaacacgtaaaaattattataatgtaataaaagctataaaaatgtcttggaaggatagatat
PcomX_DGCC12669     --aataaaaaagcaaggtaaatagccttgcttttatttattgggttatgaattctgaatattcgcataatatcgttaggtaaaacacgcaaaaattattataatgtaataaaagccataaaaatgtcttggaaggatagatat
PcomX_DGCC12691     --aataaaaaagcaaggtaaatagccttgcttttatttattgggttatgaattctgaatattcgcataatatcgttaggtaaaacacgcaaaaattattataatgtaataaaagccataaaaatgtcttggaaggatagatat
PcomX_DGCC12671     aataagtaaaagcaaggttaatagccttgcttttatttattggtttataaattctgaatattcgcataatatcgttaggtaaaacacttaaaaattattataatgaaataaaagccataaaaatgtcttgaaaggatagatat
PcomX_DGCC12668     aataagtaaaagcaaggttaatagccttgcttttatttattggtttataaattctgaatattcgcataatatcgttaggtaaaacacttaaaaattattataatgaaataaaagccataaaaatgtcttgaaaggatagatat
PcomX_DGCC12675     aataaataaaagcaaggtaaatagccttgcttttatttattgggtcataaattctgaatattcgcataatatcgttaggtaaaacacgtaaaaattattataatgtaataaaagctataaaaatgtcttggaaggatagatat
PcomX_DGCC12678     aataaataaaagcaaggtaaatagccttgcttttatttattgggtcataaattctgaatattcgcataatatcgttaggtaaaacacgtaaaaattattataatgtaataaaagctataaaaatgtcttggaaggatagatat
PcomX_DGCC12685     ----aaaaaaagcaaggtaaatagccttgcttttatttattgggttataaattctgaatattcgcataatatcgttaggtaaaacacgcaaaaattattataatgtaataaaagccataaaaatgtcttggaaggatagatat
PcomX_DGCC12651     --aataaaaaagcaaggtaaatagccttgcttttatttattgggttataaattctgaatatttgcataatatcgttaggtaaaacacgtaaaaattattataatgtaataaaagccataaaaatgtcttggaaggatagatat
PcomX_DGCC12660     --aataaaaaagcaaggtaaatagccttgcttttatttattgggttataaattctgaatatttgcataatatcgttaggtaaaacacgtaaaaattattataatgtaataaaagccataaaaatgtcttggaaggatagatat
PcomX_DGCC12686     --aataaaaaagcaaggtaaatagccttgcttttatttattgggttataaattctgaatatttgcataatatcgttaggtaaaacacgtaaaaattattataatgtaataaaagccataaaaatgtcttggaaggatagatat
PcomX_DGCC12650     --aataaaaaagcaaggtaaatagccttgcttttatttattgggttataaattctgaatattcgcataatatcgttaggtaaaacacgtaaaaattattataatgtaataaaagccataaaaatgtcttggaaggatagatat
PcomX_DGCC12653     --aataaaaaagcaaggtaaatagccttgcttttatttattgggttataaattctgaatattcgcataatatcgttaggtaaaacacgtaaaaattattataatgtaataaaagccataaaaatgtcttggaaggatagatat
PcomX_DGCC1594      --aataaaaaagcaaggtaaatagccttgcttttatttattgggttataaattctgaatattcgcataatatcgttaggtaaaacacgtaaaaattattataatgtaataaaagccataaaaatgtcttggaaggatagatat
PcomX_DGCC12662     --aataaaaaagcaaggtaaatagccttgcttttatttattgggttataaattctgaatattcgcataatatcgttaggtaaaacacgtaaaaattattataatgtaataaaagccataaaaatgtcttggaaggatagatat
PcomX_KF147         --aataaaaaagcaaggtaaatagccttgcttttatttattgggttataaattctgaatattcgcataatatcgttaggtaaaacacgcaaaaattattataatgtaataaaagccataaaaatgtcttggaaggatagatat
PcomX_DGCC12657     --aataaaaaagcaaggtaaatagccttgcttttatttattgggttataaattctgaatattcgcataatatcgttaggtaaaacacgcaaaaattattataatgtaataaaagccataaaaatgtcttggaaggatagatat
PcomX_IL1403        aataaataaaagcaaggtaaatagccttgcttttatttattgggttataaattctgaatattcgcataatatcgttaggtaaaacatgtaa-aattattataatgtaataaaagccataaaaatgtcttggaaggatagatat
PcomX_DGCC12696     aataaataaaagcaaggtaaatagccttgcttttatttattgggttataaattctgaatattcgcataatatcgttaggtgaaacatgtaaaaattattataatgtaataaaagccataaaaatgtcttggaaggatagatat
                           *********** ************************ * ** ************* ************* *** *****   *  ************* ********* ************** ** *********

PcomX_DGCC1526      gaaaatagctcattataataagatgctccctgactgtttattagatatacattctccacccc-tatcttcttactaataaatcaaataaagataatataatataatataaaaggagaactttcca     265
PcomX_DGCC12658     gaaaatagctcattataataagatgctccctgactgtttattagatatacattctccacccc-tatcttcttactaataaatcaaataaagataatataa----------aaggagaactttcca     255
PcomX_DGCC12669     gaaaatagctcattataataagatgctccctgactgtttattagatatacattcgccacccc-tatcttcttactaataaatcaaataaagataatataa----------aaggagaactttcca     255
PcomX_DGCC12691     gaaaatagctcattataataagatgctccctgactgtttattagatatacattcgccacccc-tatcttcttactaataaatcaaataaagataatataa----------aaggagaactttcca     255
PcomX_DGCC12671     gaaaatagctcattataataagatgctccctgactgtttattagacatacactcgccgccttctatcatcttactaattaattaaataaagataatataa----------aaggagaactttcca     258
PcomX_DGCC12668     gaaaatagctcattataataagatgctccctgactgtttattagacatacactcgccgccttctatcatcttactaattaattaaataaagataatataa----------aaggagaactttcca     258
PcomX_DGCC12675     gaaaatagctcattataataagatgctccctgactgtttattagatatacactcgccgccttctatcatcttactaattaatcaaataaagataatataa----------aaggagaactttcca     258
PcomX_DGCC12678     gaaaatagctcattataataagatgctccctgactgtttattagatatacactcgccgccttctatcatcttactaattaatcaaataaagataatataa----------aaggagaactttcca     258
PcomX_DGCC12685     gaaaatagctcattataataagatgctccctgactgtttattagatatacactcgccgccttctatcatcttactaattaatcaaataaagataatataa----------aaggagaactttcca     254
PcomX_DGCC12651     gaaaatagctcattataataagatgctccctgactgtttattagatatacactcgccgccttctatcatcttactaattaatcaaataaagataatataa----------aaggagaactttcca     256
PcomX_DGCC12660     gaaaatagctcattataataagatgctccctgactgtttattagatatacactcgccgccttctatcatcttactaattaatcaaataaagataatataa----------aaggagaactttcca     256
PcomX_DGCC12686     gaaaatagctcattataataagatgctccctgactgtttattagatatacactcgccgccttctatcatcttactaattaatcaaataaagataatataa----------aaggagaactttcca     256
PcomX_DGCC12650     gaaaatagctcattataataagatgctccctgactgtttattagatatacactcgccgccttctatcatcttactaattaatcaaataaagataatataa----------aaggagaactttcca     256
PcomX_DGCC12653     gaaaatagctcattataataagatgctccctgactgtttattagatatacactcgccgccttctatcatcttactaattaatcaaataaagataatataa----------aaggagaactttcca     256
PcomX_DGCC1594      gaaaatagctcattataataagatgctccctgactgtttattagatatacactcgccgccttctatcatcttactaattaatcaaataaagataatataa----------aaggagaactttcca     256
PcomX_DGCC12662     gaaaatagctcattataataagatgctccctgactgtttattagatatacactcgccgccttctatcatcttactaattaatcaaataaagataatataa----------aaggagaactttcca     256
PcomX_KF147         gaaaatagctcattataataagatgctccctgactgtttattagatatacactcgccgccttctatcatcttactaattaatcaaataaagataatataa----------aaggagaactttcca     256
PcomX_DGCC12657     gaaaatagctcattataataagatgctccctgactgtttattagatatacactcgccgccttctatcatcttactaattaatcaaataaagataatataa----------aaggagaactttcca     256
PcomX_IL1403        gaaaatagctcattataataagatgccccctgactgtttattagatatacactcgccacctcctatcaatttactaattaatcaaataaagataatataa----------aaggagaactttcca     257
PcomX_DGCC12696     gaaaatagctcattataataagatgccccctgactgtttattagatatacactcgccacctcctatcaatttactaattaatcaaataaagataatataa----------aaggagaactttcca     258
                    ************************** ****************** ***** ** ** **   ****   ******** *** *****************          ***************
```
